# Psychometric validation of a brief self-report measure of misophonia symptoms and functional impairment: The duke-vanderbilt misophonia screening questionnaire

**DOI:** 10.3389/fpsyg.2022.897901

**Published:** 2022-07-22

**Authors:** Zachary J. Williams, Carissa J. Cascio, Tiffany G. Woynaroski

**Affiliations:** ^1^Medical Scientist Training Program, Vanderbilt University School of Medicine, Nashville, TN, United States; ^2^Department of Hearing and Speech Sciences, Vanderbilt University Medical Center, Nashville, TN, United States; ^3^Vanderbilt Brain Institute, Vanderbilt University, Nashville, TN, United States; ^4^Frist Center for Autism and Innovation, Vanderbilt University, Nashville, TN, United States; ^5^Vanderbilt Kennedy Center, Vanderbilt University Medical Center, Nashville, TN, United States; ^6^Department of Psychiatry and Behavioral Sciences, Vanderbilt University Medical Center, Nashville, TN, United States; ^7^Department of Communication Sciences and Disorders, John A. Burns School of Medicine, University of Hawaii, Honolulu, HI, United States

**Keywords:** misophonia, decreased sound tolerance, screening, diagnosis, psychometric, measurement, autism, item response theory

## Abstract

Misophonia is a newly described disorder of sound tolerance characterized by strong negative emotional reactions to specific “trigger” sounds, resulting in significant distress, pathological avoidance, and impairment in daily life. Research on misophonia is still in its infancy, and most existing psychometric tools for assessing misophonia symptoms have not been extensively validated. The purpose of the current study was to introduce and psychometrically validate the duke-vanderbilt Misophonia Screening Questionnaire (DVMSQ), a novel self-report measure of misophonia symptoms that can be used to determine misophonia “caseness” in clinical and research settings. Employing large online samples of general population adults (*n* = 1403) and adults on the autism spectrum (*n* = 936), we rigorously evaluated the internal structure, reliability, validity, and measurement invariance of the DVMSQ. Results indicated that 17 of the 20 original DVMSQ items fit well to a bifactor structure with one “general misophonia” factor and four specific factors (anger/aggression, distress/avoidance, impairment, and global impact). DVMSQ total and subscale scores were highly reliable in both general population and autistic adult samples, and the measure was found to be approximately invariant across age, sex, education level, and autism status. DVMSQ total scores also correlated strongly with another measure of misophonia symptoms (Duke Misophonia Questionnaire–Symptom Scale), with correlations between these two measures being significantly stronger than correlations between the DVMSQ and scales measuring other types of sound intolerance (Inventory of Hyperacusis Symptoms [General Loudness subscale] and DSM-5 Severity Measure for Specific Phobia [modified for phonophobia]). Additionally, DVMSQ items were used to operationalize diagnostic criteria for misophonia derived from the Revised Amsterdam Criteria, which were further updated to reflect a recent consensus definition of misophonia (published after the development of the DVMSQ). Using the new DVMSQ algorithm, 7.3% of general population adults and 35.5% of autistic adults met criteria for clinically significant misophonia. Although additional work is needed to further investigate the psychometric properties of the DVMSQ and validate its theory-based screening algorithm using best-estimate clinical diagnoses, this novel measure represents a potentially useful tool to screen for misophonia and quantify symptom severity and impairment in both autistic adults and the general population.

## Introduction

Misophonia is a newly described disorder of sound tolerance in which individuals have strong negative emotional responses to specific “trigger” sounds (e.g., chewing, tapping, and sniffling), resulting in significant distress, pathological avoidance behavior, and impairment in daily life (Schröder et al., 2013; Potgieter et al., 2019; Swedo et al., 2022). When encountering a trigger sound or other non-auditory stimuli associated with such sounds (e.g., the sight of an individual eating), individuals with misophonia frequently experience emotions such as anger, extreme irritation, disgust, or anxiety, potentially combined with a “fight or flight” response and non-specific physical symptoms such as muscle tension, increased heart rate, or sweating (Edelstein et al., 2013; Rouw and Erfanian, 2018; Jager et al., 2020; Swedo et al., 2022). Other stimuli, such as purely visual triggers (e.g., a leg bouncing up and down; Schröder et al., 2013; Jaswal et al., 2021) or simply imagining a trigger sound (Ferrer-Torres and Giménez-Llort, 2021) may also be sufficient to trigger full-blown misophonic reactions in some cases. Misophonia is distinct from other forms of decreased sound tolerance such as *hyperacusis* (a disorder in which sounds of moderate intensity are perceived as excessively loud or physically painful) and *phonophobia* (a specific phobia of certain sounds or sound sources), although these different conditions may co-occur in some individuals (Fagelson and Baguley, 2018; Fackrell et al., 2019; Adams et al., 2021; Williams et al., 2021c,d; Siepsiak et al., 2022). A recent epidemiologic study using semi-structured clinical interviews estimated the prevalence of clinically significant misophonia to be 12.8% among older adolescents and adults in one urban area (Kılıç et al., 2021), additionally finding misophonia status to be associated with female sex, younger age, and multiple co-occurring psychiatric conditions. Notably, research on misophonia is in its infancy, and there is much still to be learned about the phenomenology of this condition, its underlying pathophysiology, and the most appropriate ways to screen for, diagnose, and treat misophonia in clinical practice.

As misophonia has not been formally adopted as a clinical diagnosis within existing frameworks such as the International Statistical Classification of Diseases and Related Health Problems (ICD; World Health Organization, 2019) or the Diagnostic and Statistical Manual of Mental Disorders (DSM; American Psychiatric Association, 2022), there remains a lack of consensus among stakeholders regarding the specific criteria used to determine misophonia “caseness” (i.e., the status of an individual having clinically significant misophonia) within research and clinical practice. Operational diagnostic criteria have been proposed by multiple research groups (Schröder et al., 2013; Dozier et al., 2017; Jager et al., 2020; Kılıç et al., 2021; Guetta et al., 2022), but none of these criteria were endorsed in a recent expert consensus definition of misophonia (Swedo et al., 2021, 2022). Moreover, while the published consensus definition of misophonia did deviate from existing sets of diagnostic criteria such as the Revised Amsterdam Criteria (Jager et al., 2020), the authors of the consensus statement did not publish specific diagnostic criteria in line with their definition. Therefore, in order for this foundational definition to be applied in research and practice, additional work is necessary to both (a) distil the consensus definition down to a set of diagnostic criteria and (b) operationalize these new misophonia criteria using standardized instruments such as structured interviews or questionnaires.

The present study sought to build on the newly proposed consensus definition of misophonia by providing an initial draft of updated diagnostic criteria and specific ways in which those criteria can be assessed using a published but not yet validated assessment, the duke-vanderbilt Misophonia Screening Questionnaire (DVMSQ; Williams et al., 2021a). Additionally, this study provides the first psychometric evidence supporting the reliability, latent structure, and validity of the DVMSQ as a measure of misophonia symptom severity and impairment in both general-population adults and adults on the autism spectrum, a population in which misophonia and other forms of clinically significant decreased sound tolerance are prevalent (Williams et al., 2021c,e). Although a large number of novel self-report questionnaires have recently been proposed to measure misophonia severity (Schröder et al., 2013; Wu et al., 2014; Jager et al., 2020; Siepsiak et al., 2020; Dibb et al., 2021; Remmert et al., 2021; Rinaldi et al., 2021; Rosenthal et al., 2021; Vitoratou et al., 2021), the DVMSQ differs from the majority of these measures in that it was specifically designed to operationalize the diagnostic criteria for misophonia as proposed by different authors (Schröder et al., 2013; Dozier et al., 2017; Jager et al., 2020). Further, unlike other measures, which typically assign misophonia caseness on the basis of theoretically or empirically based cutoff scores, the DVMSQ provides a criterion-based algorithm to determine whether an individual reports all symptoms and sufficient functional impairment to warrant being classified as having clinically significant misophonia. In the context of our proposed operational diagnostic criteria, derived in accord with both the Revised Amsterdam Criteria (Jager et al., 2020) and the recent consensus definition of misophonia (Swedo et al., 2022), the DVMSQ diagnostic algorithm represents the first systematic attempt to apply the misophonia consensus definition in the context of a psychometric instrument. This measure’s relative brevity, focus on theoretically “core” symptoms of the misophonia construct, and broad characterization of misophonia-related impairment suggest that the DVMSQ has potential utility as a dimensional measure of misophonia severity in both research and clinical practice.

## Methods

### Rationale and item pool development

The DVMSQ (Williams et al., 2021a) was created by the first author (ZJW) in collaboration with colleagues at the Duke Center for Misophonia and Emotion Regulation (M. Z. Rosenthal, C. Cassiello-Robbins, and D. Anand) during the development of the Duke Misophonia Questionnaire (DMQ; Rosenthal et al., 2021). While the 86-item DMQ was designed to comprehensively assess many different aspects of the misophonia construct (e.g., triggers, symptoms, cognitions, coping behaviors, beliefs, and impairment) in granular detail, the measure was not designed to assess all proposed diagnostic criteria or to discriminate between individuals with and without misophonia. Thus, the DVMSQ was created as a relatively brief complementary measure that (a) assessed diagnostic features proposed to be “core” to the misophonia construct and (b) quantified impairment due to misophonia, both in terms of interference with specific life domains and perceived global impact on one’s life.

Items on the original DVMSQ (Table 1) were adapted from the Revised Amsterdam Criteria (Jager et al., 2020), assessing the diagnostic features of (a) presence of specific “trigger” sounds, (b) intense emotional reactions (extreme irritation, anger, disgust), (c) acknowledgment that emotional reactions are excessive, (d) loss of self-control, (e) avoidance of triggers and/or endurance of triggers with distress, and (f) associated impairment due to symptoms (social, occupational, domestic, and community domains). An item about physical symptoms in reaction to triggers was additionally included to capture criterion B (i.e., the trigger stimulus elicits an immediate physical reflex response) as proposed by Dozier et al. (2017). Based on content areas represented in the broader DMQ item pool (see Rosenthal et al., 2021 for more details), items were also added to assess (a) fear or panic in response to triggers, (b) attentional capture by trigger sounds, and (c) perceived global impact (including negative effects on mental health). Notably, while specific efforts were made during the development of the DMQ to separate “double-barreled” items, items of the DVMSQ were written to be more general, often combining multiple related emotions or sensations into single items for the sake of brevity and to reduce local item dependence. All items were initially drafted by ZJW, and wording was iteratively refined until consensus was achieved. As an important caveat, the development and finalization of the DVMSQ occurred before the initial publication of the misophonia consensus definition (Swedo et al., 2021), and thus, not all aspects of the condition mentioned in the consensus definition and proposed diagnostic criteria are included in the DVMSQ. Nevertheless, as existing DVMSQ items operationalize all but one of the proposed diagnostic criteria (symptom duration ≥6 months), the measure represents a reasonable approximation of the misophonia construct as recently defined.

**TABLE 1:** Original DVMSQ items, content, and response options.

Item	Verbatim content	Response options
S1	Are there specific sounds that you are extremely bothered by, even if they are not loud? *Examples include: chewing, slurping, crunching, throat clearing, finger tapping, foot shuffling, keyboard tapping, rustling, nasal sounds, pen clicking, appliance humming, clock ticking, and animal sounds.*	Yes/No
S2	Please list the sounds that you are extremely bothered by, even when they are soft.	[Free Text]
	**When you are exposed to the bothersome sounds listed above, how often do you experience.**	
1	Intense feelings of irritation or annoyance?	Never/Rarely/Sometimes/Often/Very often
2	Feelings of anger or rage?	Never/Rarely/Sometimes/Often/Very often
3	Feelings of fear or panic?	Never/Rarely/Sometimes/Often/Very often
4	Feelings of disgust?	Never/Rarely/Sometimes/Often/Very often
5	Urges to run away from the sound?	Never/Rarely/Sometimes/Often/Very often
6	Urges to cover your ears or block out the sound in some other way?	Never/Rarely/Sometimes/Often/Very often
7	Urges to lash out violently at the person or object making the sound?	Never/Rarely/Sometimes/Often/Very often
8	Feeling like you cannot control your response to the sound?	Never/Rarely/Sometimes/Often/Very often
9	Difficulty focusing on anything except the sound?	Never/Rarely/Sometimes/Often/Very often
10	Some sort of immediate physical response? *(e.g., tensing of muscles, heart racing, warmth, tingling, pain, or tightening of stomach)*	Never/Rarely/Sometimes/Often/Very often
10b	Please describe the immediate physical response you have to the above sounds.	[Free Text]
11	How often are your emotional responses to these bothersome sounds excessive, unreasonable, or out of proportion to how most other people would respond?	Never/Rarely/Sometimes/Often/Very often
12	How often do you avoid situations where you may potentially hear these bothersome sounds?	Never/Rarely/Sometimes/Often/Very often
	**In the past 7 days, how much did your sound sensitivities interfere with.**	
13	Your ability to interact with other people?	Not at all/A little bit/A moderate amount/Very much/An extreme amount
14	Your ability to be productive at work or school?	Not at all/A little bit/A moderate amount/Very much/An extreme amount
15	Your ability to take care of your household responsibilities?	Not at all/A little bit/A moderate amount/Very much/An extreme amount
16	Your ability to participate in community activities (for example, festivities, religious, or other activities)?	Not at all/A little bit/A moderate amount/Very much/An extreme amount
17	Your ability to concentrate?	Not at all/A little bit/A moderate amount/Very much/An extreme amount
18	To what degree have your sound sensitivities negatively impacted your mental or emotional health?	Not at all/A little bit/A moderate amount/Very much/An extreme amount
19	To what degree do you believe that your sound sensitivities have created problems for you?	Not at all/A little bit/A moderate amount/Very much/An extreme amount
20	To what degree do you believe that your sound sensitivities have made your entire life worse?	Not at all/A little bit/A moderate amount/Very much/An extreme amount

### Operational diagnostic criteria for misophonia

In order to create operational diagnostic criteria that could be assessed using the DVMSQ, we began with the Revised Amsterdam Criteria (Jager et al., 2020), modifying each criterion to align more closely with the recent misophonia consensus definition (Swedo et al., 2021, 2022). The misophonia diagnostic criteria used in the current study (see also Williams, 2022) are presented in Table 2, along with the operationalization of each criterion by the DVMSQ items. Notable changes from the Revised Amsterdam criteria include (a) the removal of the requirement that an individual be triggered by oral or nasal sounds, (b) the removal of the requirement that individuals must acknowledge their emotional reactions to triggers as excessive, unreasonable, or out of proportion to the circumstances, (c) additional description of anxiety and/or physical symptoms accompanying the emotional reactions (though neither is required for diagnosis nor sufficient to fulfill that criterion), (d) the use of specific coping strategies (e.g., ear protection, masking trigger sounds with white noise) is described within the “avoidance” criterion, (e) the outbursts resulting from a loss of control are described in more detail and include manifestations other than aggression, and (f) emotional reactions occurring in the context of other neuropsychiatric conditions (e.g., autism, ADHD) can still count toward a diagnosis of misophonia if the remaining criteria are met. Additionally, to clarify the chronic nature of misophonia symptoms, the newly proposed diagnostic criteria include a duration criterion of 6 months or longer, on par with the criteria used to diagnose most anxiety disorders (American Psychiatric Association, 2022). Although these operational criteria are designed to reflect the consensus definition of misophonia more closely than the Revised Amsterdam criteria, it is important to note that they were not themselves derived from expert consensus or a similarly rigorous Delphi process (Niederberger and Spranger, 2020). Thus, while our study does represent the first attempt to derive a diagnostic algorithm that incorporates the misophonia consensus definition, the specific criteria proposed should be treated as provisional and superseded by more rigorously developed “consensus diagnostic criteria” for misophonia as soon as such criteria are made available.

**TABLE 2:** Operational diagnostic criteria for misophonia and DVMSQ-based assessment.

Criterion	DVMSQ operationalization
A. Presence of one or more commonplace “trigger” sounds*^a^* that reliably elicit intense and inappropriate emotional responses, irrespective of sound intensity or perceived loudness.	**Item S1 [Screening] = Yes**
B. Trigger sounds reliably*^b^* evoke feelings of extreme irritation, anger, rage and/or disgust*^c^* that are clearly excessive, unreasonable, or out of proportion to the circumstances (whether or not the individual recognizes them as such).	One or more of the following: **Item 1 [Irritation] ≥ Often** **Item 2 [Anger/Rage] ≥ Often** **Item 4 [Disgust] ≥ Often**
C. The individual actively avoids*^d^* situations or activities that include trigger sounds, endures these situations with intense discomfort, or needs to block out potential trigger sounds (e.g., using earplugs, music, or white noise) to cope with these situations.	One or more of the following: **Item 12 [Avoidance] ≥ Sometimes** **Item 5 [Urge to run away] ≥ Often** **Item 6 [Urge to cover ears] ≥ Often**
D. If unable to avoid trigger sounds or stop them from occurring, the individual experiences a significant loss of self-control, potentially resulting in emotional outbursts or other extreme reactions (e.g., yelling/screaming, running out of the room, panic attacks, and rarely physical aggression).	One or more of the following: **Item 8 [Lack of Control] ≥ Sometimes** **Item 7 [Urge to be violent] ≥ Sometimes**
E. The emotional reactions to trigger sounds are persistent, typically lasting for 6 months or more. Specific triggers do not need to remain constant over this period, but at least one trigger sound must meet both criteria A and B at all times over the preceding 6-month period.	Not assessed by DVMSQ Assumed to be true if all other criteria are satisfied.
F. Emotional reactions to trigger sounds and/or avoidance of these sounds cause clinically significant distress or impairment in social, occupational, or other important areas of functioning.	Two or more of the following: **Item 13 [Social] ≥ Moderate** **Item 14 [Occupational] ≥ Moderate** **Item 15 [Household] ≥ Moderate** **Item 16 [Community] ≥ Moderate** **Item 18 [Mental Health] ≥ Moderate** **Item 19 [Global Problems] ≥ Moderate** **Item 20 [Life Affected] ≥ Moderate**

To meet the duke-vanderbilt Misophonia Screening Questionnaire (DVMSQ) criteria for “clinically significant misophonia,” an individual must meet all criteria A–F (criteria E is not assessed by the DVMSQ and is assumed to be true if all others are satisfied). Individuals who meet criteria A–D but not criterion F are classified as having “sub-clinical misophonia.” Note that the item numbers in this algorithm refer to the original DVMSQ and not the revised version provided in the supplemental information.

^a^In accordance with the recent consensus definition of misophonia, these criteria do not require that the individual be triggered by chewing or other oro-nasal sounds.

^b^These emotional reactions may be dependent on the context in which the trigger is encountered (e.g., only occurring when the trigger is produced by a specific person), but the reaction should be easily reproducible within that specific context.

^c^Emotional responses to trigger sounds may be accompanied by fear, anxiety, or physical symptoms of sympathetic arousal (e.g., heart pounding, muscle tension, sweating, and paresthesia), but in the absence of anger, irritation or disgust, these reactions are insufficient to meet criterion B.

^d^Includes both direct avoidance of the trigger stimulus and indirect avoidance (i.e., actions taken to stop the stimulus from occurring, such as telling another person to stop making a sound, removing triggering household items, etc.).

### Participants

The current study comprises a secondary data analysis of two large survey studies that included the DVMSQ as a part of longer survey batteries assessing multiple types of decreased sound tolerance (i.e., hyperacusis, misophonia, and phonophobia) as well as their clinical and demographic correlates in adults. The primary sample analyzed in this investigation is a large online general-population sample of adults in the United States (*n* = 1403) recruited from the Prolific crowdsourcing platform (Palan and Schitter, 2018; Stanton et al., 2022). Additionally, in order to assess the psychometric properties of the DVMSQ in autistic adults, we examined data from a sample of independent autistic adults (*n* = 936) from the Simons Foundation Powering Autism Research for Knowledge (SPARK) cohort (Feliciano et al., 2018). Notably, data from the SPARK sample were predominantly included to assess the latent structure of the DVMSQ in the autistic population and to examine differential item functioning across diagnostic groups; thus, the majority of analyses in the current study focus exclusively on the general population (Prolific) sample.

#### General population (Prolific) sample

A sample of general population adults was recruited from the Prolific crowdsourcing platform (Palan and Schitter, 2018; Stanton et al., 2022) in the fall of 2021. Eligibility criteria included age 18 or older, living in the United States, speaking English fluently, having answered Prolific demographic questions about autism status and current mental health conditions (any non-missing response to both questions was sufficient for inclusion), not endorsing a diagnosis of dementia or mild cognitive impairment, having completed at least 50 previous Prolific tasks, and a 95% or higher approval rate on Prolific. Individuals endorsing severe/profound hearing loss or the use of cochlear implants were also excluded from the current study *post hoc*. Participants were recruited in two single-sex batches of 750 (i.e., 750 males and 750 females, recruited concurrently) to ensure approximate sex parity. The study survey was advertised as examining “sensory sensitivities,” although participant-facing materials did not specify that the study investigated decreased sound tolerance or misophonia specifically. The full study survey included questionnaires on demographics, medical/psychiatric history, decreased sound tolerance symptoms, other sensory experiences, personality traits, psychopathology, somatic symptom burden, and overall quality of life, and surveys were completed on the REDCap platform (Harris et al., 2009). All participants gave their informed consent for the study, and participants who completed the Prolific task were compensated $5.00 USD for their time. All study procedures were approved by the Institutional Review Board at Vanderbilt University Medical Center.

In order to ensure that the results of the Prolific survey were of high quality, a rigorous data quality assessment was undertaken to flag and remove potentially invalid responses (Chandler et al., 2020). Participants were excluded if they (a) failed one or more directed-response attention check questions embedded within the survey (e.g., “To show that you are paying attention, please leave this question blank”), (b) endorsed a diagnosis of Alzheimer’s disease or dementia despite denying that diagnosis on their Prolific demographics, (c) endorsed one or more “infrequency” items in the medical history (e.g., a reported history of temporal lobectomy), (d) provided symptom information that was inconsistent with their lifetime medical diagnoses (e.g., endorsed migraines caused by sound but denied experiencing migraines), (e) reported information about their demographics or autism diagnostic status that was inconsistent with the information provided on their Prolific demographics form, (f) completed the survey in an exceptionally short amount of time (i.e., more than three median absolute deviations below the median completion time), or (g) endorsed random or dishonest responding when queried at the end of the survey with “no penalty honesty check” questions (e.g., “*Did you answer any survey questions in this survey randomly? Your answer will not affect your compensation for this survey.*”). Participants were also excluded if they completed the survey from a virtual private network, an IP address located outside of the United States, or an IP address associated with multiple survey respondents. Of the 1610 individuals who consented for the study, 1516 individuals completed the full Prolific survey and had their submissions approved. Of these participants, 113 individuals (7.5%) were excluded for failing one or more data quality check, leaving a final sample of 1403 individuals whose data were analyzed for the current study (note that not all 1403 individuals were included in all analyses).

#### Autistic (SPARK) sample

A sample of legally independent autistic adults was recruited from the SPARK Research Match service (Project No. RM0111Woynaroski_DST). A largely overlapping sample has previously been described elsewhere (Williams et al., 2022). Eligibility criteria included age 18 or older, self-reported professional diagnoses of autism spectrum disorder or equivalent conditions (e.g., Asperger syndrome, Pervasive Developmental Disorder–Not Otherwise Specified), and legal independence (i.e., ability to consent for oneself). Although autism diagnoses were not independently confirmed, prior research has generally supported the validity of self-reported autism diagnoses within the SPARK cohort (Fombonne et al., 2021). These participants completed a series of online surveys assessing demographics, medical/psychiatric history, core features of autism, co-occurring psychopathology, somatic symptom burden, and quality of life, and the instruments administered partially overlapped with those in the Prolific study. Surveys were completed within the SPARK web platform using custom software designed by Tempus Dynamics (Baltimore, MD, United States), and participants were compensated with $10 USD in Amazon gift cards upon completion of all surveys. All participants gave informed consent, and all study procedures were approved by the institutional review board at Vanderbilt University Medical Center.

Individuals in the SPARK sample additionally underwent a series of similar quality checks to the Prolific sample. Specifically, survey participants from the SPARK RM sample were excluded if they (a) met the SPARK definition of a possibly invalid autism diagnosis (e.g., age of diagnosis is under 1 year of age; diagnosis rescinded by a professional), (b) did not self-report a professional diagnosis of autism on the study-specific demographics form, (c) reported demographic variables (e.g., age, sex at birth, receipt of special education services in childhood) that were inconsistent with those originally reported to SPARK, (d) reported the use of a cochlear implant, or (e) endorsed a professional diagnosis of either Alzheimer’s disease or dissociative identity disorder (indicating either careless/random responding or a true diagnosis that could compromise the validity of self-report). Additionally, individuals who dropped out of the study before completing the DVMSQ and other sound tolerance measures were not included in the current analyses. Of the 1271 individuals who initially consented for the study, 1121 completed the measures of interest. Of these individuals, an additional 185 (16.5%) were excluded after failing one or more data quality checks, leaving a final sample of 936 autistic adults from SPARK whose data were analyzed in the current study.

### Measures

#### Duke-vanderbilt misophonia screening questionnaire

The duke-vanderbilt misophonia Screening Questionnaire (DVMSQ; Williams et al., 2021a) is a brief self-report measure designed to assess the symptoms of misophonia proposed in the Revised Amsterdam Criteria (Jager et al., 2020), as well as functional impairment due to misophonia. The measure also includes additional associated symptoms found to be potentially relevant during the item-generation process for the DMQ, including trigger-evoked fear/panic, physical symptoms, and attention capture by the trigger stimulus. The version of the DVMSQ administered to the Prolific and SPARK cohorts contained 21 items (one Yes/No “screening” item and 20 Likert items), as well as two free-text fields to allow participants to expand upon their trigger sounds and trigger-evoked physical symptoms, respectively (see Table 1 for full item content). Respondents are first asked a single screening question (“*Are there specific sounds that you are extremely bothered by, even if they are not loud? Examples include: chewing, slurping, crunching, throat clearing, finger tapping, foot shuffling, keyboard tapping, rustling, nasal sounds, pen clicking, appliance humming, clock ticking, and animal sounds.*”), and if they respond “No” to this question, no further DVMSQ items are administered. For participants who answer the screening question affirmatively, they are presented with a free-text field in which they are asked to list their specific trigger sounds. The remaining questions include 12 “symptom frequency” items (rated on a 5-point Likert scale from 0 = “*Never*” to 4 = “*Very often*”), as well as 8 “impairment” items (rated on a 5-point Likert scale from 0 = “*Not at all*” to 4 = “*An extreme amount*”). A subset of these items is additionally used to operationalize the misophonia diagnostic criteria presented in this study (see Table 2 for specifics). Scores on the 20 DVMSQ symptom and impairment items were examined in the psychometric analysis of the current study.

#### Duke misophonia questionnaire

The Duke Misophonia Questionnaire (DMQ; Rosenthal et al., 2021) is an 86-item modular self-report questionnaire that assesses a wide range of misophonia-related constructs, including specific triggers, trigger frequency, responses to misophonic triggers (affective, physiological, and cognitive), specific coping strategies (before, during and after being triggered), misophonia-related impairment, and dysfunctional beliefs related to misophonia. This measure was rigorously developed using an iterative item generation process with suggestions and feedback directly from individuals with misophonia and their families, and a preliminary psychometric study has established the latent structure, reliability, and convergent validity of the DMQ subscales in a sample of general-population adults (Rosenthal et al., 2021). In order to reduce participant burden in the Prolific and SPARK surveys, participants completed an abbreviated version of the DMQ that included only (a) the trigger list (16 Yes/No items), (b) the “frequency of being triggered” item (6-point Likert scale from 1 = “*Once per month or less*” to 6 = “*6 or more times per day*”), (c) the 23 DMQ symptom scale (DMQ-SS) items (5 affective, 8 physiological, 10 cognitive; rated on a 5-point Likert scale from 0 = “*Not at all*” to 4 = “*Always/Almost always*”), and a novel “global impairment” item (“*Please rate the overall impact of ALL bothersome sounds on your life over the past month.*”) that was rated on a visual analog scale (VAS) ranging from 0 = “*No Effect”* to 100 = “*Extreme Effect.*” SPARK participants also completed the DMQ Impairment Scale (12 items), but, given the focus of the current investigation of this study on the Prolific sample, this scale was not examined in our analyses. Participants who did not endorse any triggers on the trigger list did not complete the remaining DMQ questions. Measures derived from the DMQ included (a) number of trigger categories endorsed (range 0–15), trigger frequency (range 1–6), DMQ-SS mean symptom score (range 0–4), and global impairment VAS (range 0–100). The reliability of the DMQ-SS in the Prolific sample was excellent (α = 0.946), and the DMQ-SS correlated strongly with all other DMQ-derived variables (number of trigger categories: *r* = 0.545, CI_95%_ [0.501, 0.587]; trigger frequency: *r*_*poly*_ = 0.635, CI_95%_ [0.597, 0.670]; global impairment VAS: *r* = 0.625, CI_95%_ [0.586, 0.661]).

#### Inventory of hyperacusis symptoms

The Inventory of Hyperacusis Symptoms (IHS; Greenberg and Carlos, 2018) is a 25-item self-report questionnaire designed to assess the symptoms of hyperacusis, as well as emotional responses to sounds, quality of life, mental health impact, and functional impairment due to decreased sound tolerance. Items are organized into five empirically derived subscales, including general loudness (3 items), emotional arousal (6 items), psychosocial impact (9 items), functional impact (5 items), and communication (2 items). This measure has demonstrated strong reliability, as well as some degree of convergent/divergent validity in both an online sample of individuals with tinnitus and/or hyperacusis (Greenberg and Carlos, 2018) and a care-seeking sample of individuals attending a specialist tinnitus and hyperacusis clinic in the United Kingdom (Aazh et al., 2021). Although designed to specifically assess hyperacusis, the IHS has not been formally tested in individuals with misophonia or other sound tolerance disorders; thus, it is unclear the degree to which the IHS subscales measuring emotional arousal and psychosocial/functional impact are confounded by misophonia severity. Thus, while the IHS total score (range 25–100) was reported descriptively as a measure of “hyperacusis severity” in the current study, the “general loudness” subscale (IHS-LOUD; range 3–12) was examined in analyses of discriminant validity due to its lack of content overlap with misophonia measures such as the DVMSQ and the DMQ. In the Prolific sample, reliability was good for both the IHS total score (α = 0.963) and the IHS-LOUD score (α = 0.803).

#### DSM-5 severity measure for specific phobia–modified for phonophobia

The DSM-5 Severity Measure for Specific Phobia (DSM-SP; Lebeau et al., 2012) is a 10-item scale published by the American Psychiatric Association to dimensionally assess symptoms of specific phobias in adults. Participants are first asked to determine which of five common phobia topics (e.g., “*Animals or insects*”; “*Blood, needles, or injections*”) is most anxiety provoking for them, proceeding to rate their symptoms over the past week when encountering situations related to the topic chosen. Items are rated on a 5-point Likert scale with responses ranging from 0 = “*Never*” to 4 = “*All of the time*,” and the mean score (range 0–4) is calculated as a dimensional index of phobia severity. The reliability and validity of the DSM-SP has been established in both clinical and non-clinical adult samples (Lebeau et al., 2012; Knappe et al., 2013, 2014). In the current study, this measure was modified to specifically assess *phonophobia* rather than other specific phobias. Thus, in the current study, we omitted the choice of phobic topics from the DSM-SP and instead administered the items with the following instructions: “*The following questions ask about thoughts, feelings, and behaviors that you may have had in a variety of situations. Over the PAST SEVEN DAYS, how often have you experienced the following regarding situations when you are exposed to loud or unpleasant sounds?*” The wording of the DSM-SP questions themselves was unchanged from the original version. In the Prolific sample, the reliability of the DSM-SP (with directions modified as detailed above) was excellent (α = 0.925).

#### Additional measures of psychopathology, somatic symptoms, and quality of life

Several additional self-report questionnaires (administered to the Prolific sample only) were collected in order to assess the nomological validity of the DVMSQ. Symptoms of general anxiety and depression were measured using the Overall Anxiety Severity and Impairment Scale (OASIS; Norman et al., 2006) and Overall Depression Severity and Impairment Scale (ODSIS; Bentley et al., 2014; Ito et al., 2015), respectively. Possible scores on these measures range from 0 to 20, and reliabilities in the Prolific sample were good (OASIS: α = 0.862; ODSIS: α = 0.871). Clinically relevant manifestations of anger (including subjective feelings of anger, overt verbal aggression, and destructive urges) in the past week were assessed using the Clinically Useful Anger Outcome Scale (CUANGOS; Levin-Aspenson et al., 2021). CUANGOS scores range from 0 to 20, and reliability in the Prolific sample was good (α = 0.857). Multi-system somatic symptom burden was measured using the Somatic Symptom Scale–8 (SSS-8; Gierk et al., 2014). SSS-8 total scores range from 0 to 32, and this score exhibited good reliability in the Prolific sample (α = 0.814). Lastly, overall quality of life (i.e., general life satisfaction) was measured using the 6-item Riverside Life Satisfaction Scale (RLSS; Margolis et al., 2019). RLSS total scores range from 6 to 42, and reliability in the Prolific sample was excellent (α = 0.900).

### Statistical analyses

All statistical analyses were conducted in R version 4.1.0 (R Core Team, 2021). Relevant demographic and clinical variables were summarized descriptively. Differences between the Prolific and SPARK samples on demographic and clinical variables were quantified using Cohen’s *d* for continuous variables and odds ratios (ORs) for categorical variables.

#### Duke-vanderbilt misophonia screening questionnaire item analysis

Item-level statistics, including category endorsement frequencies, percent endorsement of each item (at a level fulfilling the operational diagnostic criteria), and corrected item-total (polyserial) correlations, were examined for all DVMSQ participants within the Prolific sample who affirmatively answered the DVMSQ screening question (*n* = 833). We additionally calculated the polyserial correlation between each DVMSQ item and scores on (a) the DMQ-SS (misophonia symptoms), (b) the DSM-SP (phonophobia symptoms), and (c) the IHS-LOUD (hyperacusis symptoms), with the hypothesis that items measuring misophonia *symptoms* (though not necessarily items measuring misophonia-related impairment) would correlate more strongly with the DMQ-SS than with either the DSM-SP or IHS-LOUD.

#### Structural analyses of the duke-vanderbilt misophonia screening questionnaire

In order to assess the overall dimensionality of the DVMSQ, we first performed an exploratory graph analysis (EGA; Golino and Epskamp, 2017; Golino et al., 2020) using the *EGAnet* R package (Golino and Christensen, 2020). The EGA was performed using a regularized partial correlation network based on polychoric correlations (“EBICglasso” estimation with γ = 0.5 and λ = 0.1), and communities were determined using the Walktrap algorithm with four steps (Christensen et al., 2020). In order to better approximate the normal latent trait distributions assumed by the polychoric correlations, the analysis was performed on only data from individuals who completed all DVMSQ items (i.e., the zeros in the zero-inflated distribution were discarded). Although the number of dimensions was the primary variable of interest derived from this analysis, we also investigated the community assignment of the various DVMSQ items, determining whether this process identified communities that conformed to the theoretical dimensions of symptoms and impairment. In cases where specific items (particularly those not reflecting the operational diagnostic criteria for misophonia) were not clustering as expected with other items (i.e., an impairment item being assigned to a symptom dimension or vice versa), those items were removed from the analysis, and the EGA process was repeated. To further assess the dimensionality of the DVMSQ, we additionally employed the Factor Forest method (Goretzko and Bühner, 2020, 2022), a novel machine-learning based factor retention criterion that has shown excellent performance in recent simulation studies.

After assessing the dimensionality of the full DVMSQ, we investigated the latent structure of the symptom and impairment dimensions separately using full-information bifactor item response theory (IRT) modeling with iterative model refinement and replication in a holdout sample. Individuals who did not affirm the DVMSQ screening question (and therefore did not fill out all subsequent items referring to one’s experience of triggering sounds) were excluded from IRT analysis. To perform our IRT analyses, the 833 individuals in the Prolific sample who answered all DVMSQ questions were divided into exploratory (*n* = 417) and confirmatory [holdout] (*n* = 416) subsamples. A bifactor graded response model (Gibbons et al., 2007; Cai et al., 2011; Toland et al., 2017) was fit to item response data for symptoms and impairment items separately in the exploratory subsample, with results from the EGA community assignment used to preliminarily assign items to specific factors. Models were fit using the *mirt* R package (Chalmers, 2012), with the Bock and Aitkin (1981) Expectation-Maximization algorithm employed for models without cross-loadings on specific factors and the Quasi-Monte Carlo Expectation-Maximization algorithm (Hori et al., 2020) employed for all other models.

Global IRT model fit was assessed using the limited-information *C*_2_ fit index (Cai and Monroe, 2014; Monroe and Cai, 2015) accompanied by *C*_2_-based approximate fit indices, including the Tucker-Lewis index (TLI_*C*2_; Cai et al., 2021), root mean square error of approximation (RMSEA_*C*2_; Maydeu-Olivares and Joe, 2014), and standardized root-mean-square residual (SRMR; Maydeu-Olivares and Joe, 2014). Local model misfit was assessed based on examination of standardized residuals, with | *r*_*res*_| > 0.1 judged to indicate significant model misspecification. Additionally, local item dependence was evaluated using the Q_3_ residual correlation (Yen, 1984), with model-specific critical values based on the 99th percentile of a simulated distribution (1,000 simulated datasets) based on parametric bootstrapping (Christensen et al., 2017). Within the exploratory subsample, items that demonstrated either | *r*_*res*_| > 0.1 or Q_3_ values above the empirical cutoff value were either deleted from the model or specified to load onto another specific factor. Additionally, when an item loaded poorly onto a specific factor (i.e., standardized | λ| < 0.1), that factor loading was set to zero in future model iterations. This process was repeated until the final exploratory models for symptoms and impairment demonstrated no significant local misspecification and all specific factor loadings were greater than 0.1. The exploratory model was then re-fit in the holdout sample, and global/local misfit were evaluated using the same criteria. Final models for symptoms and impairment were then re-fit in the combined Prolific sample (*n* = 833), and final model parameters were examined. Bifactor indices (Rodriguez et al., 2016) were also examined to evaluate each dimension’s model-based reliability (omega total [ω_*T*_]), general factor saturation (omega hierarchical [ω_*H*_]), and essential unidimensionality (explained common variance [ECV]).

Once psychometrically adequate models were chosen for both symptoms and impairments, the two scale subsections were fit within a single bifactor IRT model. This model was evaluated in the exploratory sample for potential misspecification, with a slightly more relaxed misspecification criterion of | *r*_*res*_| > 0.15 used to accommodate the larger model size and substantially increased number of residual correlations. Misspecifications were addressed iteratively, and the final model fit was tested in the hold-out sample for confirmation. Once an adequate model was generated for the full DVMSQ, this model was fit in the combined Prolific sample (*n* = 833), and final model parameters (including model-based total score reliability [ω_*T*_], general factor saturation [ω_*H*_], and essential unidimensionality [ECV]) were examined. This final model generated in the Prolific sample was then re-fit in the SPARK sample (again using only the individuals who completed all DVMSQ items; *n* = 645) and evaluated for global and local misspecification to determine whether the structure of the DVMSQ was configurally invariant across the two populations. Model parameters and bifactor indices in the autistic sample were also examined.

Once a structural model was found to adequately fit both the Prolific and SPARK samples, we fit multiple-group IRT models to the full dataset, which were then used to test differential item functioning (DIF) of the DVMSQ items between diagnostic groups. For the purpose of DIF analyses, individuals in the Prolific sample who self-reported an autism diagnosis were considered as belonging to the autism group. DIF testing was performed using a version of the iterative Wald test procedure proposed by Cao et al. (2017), in which all items are tested for DIF using a “Wald-2” procedure (Woods et al., 2013), all items not demonstrating DIF are selected as anchors, and the Wald test is performed again on the remaining items iteratively until all tested items show DIF at the *p* < 0.05 level (uncorrected). Given the likelihood of this method to detect trivially small yet non-zero DIF at the sample sizes tested in the current study (Williams et al., 2021b; Williams and Gotham, 2021a,b), items with a standardized DIF effect size (expected score standardized difference [ESSD]; Meade, 2010) less than ±0.2 (i.e., smaller than Cohen’s (1988) definition of a “small” effect) were also included as anchor items for the iterative Wald procedure. In order to reduce computational burden and address model convergence issues, means and variances of all specific factors (i.e., all factors except the general factor) were fixed to the values used in the “Wald-2” procedure. This version of the iterative Wald method was implemented using a custom R function written by the first author (Williams, 2021). An item was flagged as exhibiting practically significant DIF if both (a) the omnibus Wald test demonstrated a *p*-value < 0.05 after Benjamini-Hochberg false discovery rate correction (i.e., *p*_*FDR*_ < 0.05) and (b) the ESSD effect size was greater than 0.5, indicating a “medium” or larger amount of DIF (Meade, 2010). Moreover, the combined biasing effect of all DIF on sum score differences between groups (i.e., differential test functioning [DTF]) was evaluated in total score units (UETSDS) and Cohen *d* units (ETSSD), with values of ETSSD > 0.1 judged to be a practically meaningful amount of DTF. Significant omnibus Wald tests demonstrating DIF in individual items were followed up with separate tests of slopes vs. intercept differences in order to determine whether DIF was uniform (affecting intercepts only) or non-uniform (affecting slopes with or without interceptions) (Stover et al., 2019). In addition to assessing DIF by diagnostic group, we also examined DIF within the Prolific sample according to age (<30 vs. ≥30), sex (Female vs. Male), and level of education (any college degree vs. “some college” or less). Notably, the cutpoints used to dichotomize age and education were chosen to allow for sufficiently large numbers of participants (i.e., >300) in each subgroup, increasing the measurement precision in the focal group and power to detect significant DIF.

#### Validity testing

After evaluating the latent structure and DIF of the DVMSQ, we calculated summary scores for the measure, including a total score (17 items; range 0–68), symptom score (10 items; range 0–40), and impairment score (7 items; range 0–28). To assess the nomological validity of the DVMSQ-derived scores, we examined zero-order Pearson correlations between DVMSQ scores and correlates of interest (i.e., scores from the DMQ, IHS, DSM-SP, OASIS, ODSIS, CUANGOS, SSS-8, and RLSS) in both the whole Prolific sample (*n* = 1403) and the subsample that completed all DVMSQ items (*n* = 833). We hypothesized that the DVMSQ scores would exhibit strong positive correlations (*r* > 0.5) with all DMQ-derived scores (based on the minimal accepted criteria for convergent validity; Carlson and Herdman, 2012), as well as moderate positive correlations with all remaining variables (*r* > 0.3) except for the RLSS score, which was expected to demonstrate a moderate negative correlation with the DVMSQ (*r* < −0.3). To further demonstrate the construct validity of the DVMSQ scores, we used the Zou (2007) confidence interval procedure to test whether the three DVMSQ-derived scores correlated more highly with the DMQ-SS than with measures of other types of decreased sound tolerance (IHS-LOUD, DSM-SP), anxiety (OASIS), depression (ODSIS), or somatic symptoms (SSS-8). Moreover, given the central role that anger plays in the construct of misophonia, we further hypothesized that the DVMSQ would correlate more highly with the CUANGOS score than with either the OASIS or ODSIS. All comparisons between dependent correlations were conducted using the *cocor* R package (Diedenhofen and Musch, 2015).

## Results

### Participant characteristics

In total, the combined sample included DVMSQ data from 2339 individuals across the two data sources, 1478 of whom (Prolific: *n* = 833; SPARK: *n* = 645) affirmatively answered DVMSQ screening question S1 and went on to complete the full measure. Demographics and clinical characterization of each sample (as well as the portions of the sample who (a) had screen-positive responses to DVMSQ item S1 [“S1 Positive” group] and (b) met the DVMSQ definition of misophonia [“Clinical Misophonia” group]) are presented in Table 3. Adults in the current study ranged from 18 to 83 years old, with participants in the SPARK sample (mean [*SD*] age = 37.49 [13.28] years) being slightly older on average than those in the Prolific sample (mean [*SD*] age = 32.27 [12.55] years), *d* = 0.41, CI_95%_ [0.32, 0.49]. Though the Prolific sample had a balanced sex ratio by design (51.1% female sex among retained participants), this was not the case for the SPARK sample (63.0% female sex), which contained a significantly higher proportion of participants assigned female at birth, OR = 1.63, CI_95%_ [1.38, 1.93]. Non-Hispanic White participants made up the majority of individuals in both samples (Prolific: 70.4%; SPARK: 80.1%), and approximately half of participants in each sample had completed a 4-year college degree (Prolific: 50.4%, SPARK: 48.2%). The median age of autism diagnosis in the SPARK sample was 23.21 years (IQR [11.77, 36.79]), with 38.7% of the sample being diagnosed with autism before the age of 18. Notably, an additional 32 individuals from the Prolific sample (2.3% of total sample, 43.8% female, mean [*SD*] age = 32.22 [12.14] years) reported receiving professional diagnoses of autism at a median age of 22.50 years (IQR [15.75, 32.50], range 3–50 years).

**TABLE 3:** Demographic and clinical characteristics of Prolific and SPARK samples.

	Prolific sample			SPARK sample		
	Full sample	S1 positive	Clinical misophonia	Full sample	S1 positive	Clinical misophonia
Sample size	1403	833	102	936	647	332
Age (years)	32.27 (12.55)	31.11 (11.91)	29.69 (10.97)	37.49 (13.28)	37.44 (12.82)	37.19 (12.40)
Sex						
Male	686 (48.9%)	334 (40.1%)	28 (27.5%)	346 (37.0%)	184 (28.4%)	71 (21.4%)
Female	717 (51.1%)	499 (59.9%)	74 (72.5%)	590 (63.0%)	463 (71.6%)	261 (78.6%)
Gender						
Male	669 (47.7%)	321 (38.5%)	27 (26.5%)	283 (36.2%)	150 (27.7%)	55 (19.9%)
Female	685 (48.8%)	468 (56.2%)	64 (62.7%)	457 (58.5%)	356 (65.8%)	194 (70.3%)
Non-binary or other gender	49 (3.5%)	44 (5.3%)	11 (10.8%)	41 (5.2%)	35 (6.5%)	27 (9.8%)
Race						
White	1106 (78.8%)	674 (80.9%)	85 (83.3%)	854 (91.2%)	592 (91.5%)	307 (92.5%)
American indian or alaska native	26 (1.9%)	14 (1.7%)	3 (2.9%)	57 (6.1%)	46 (7.1%)	30 (9%)
Asian	154 (11%)	80 (9.6%)	3 (2.9%)	38 (4.1%)	23 (3.6%)	16 (4.8%)
Black or african american	104 (7.4%)	59 (7.1%)	9 (8.8%)	40 (4.3%)	31 (4.8%)	17 (5.1%)
Middle eastern or north african	20 (1.4%)	12 (1.4%)	3 (2.9%)	8 (0.9%)	7 (1.1%)	5 (1.5%)
Native hawaiian or other pacific islander	3 (0.2%)	2 (0.2%)	0 (0%)	4 (0.4%)	3 (0.5%)	3 (0.9%)
Other race	8 (0.6%)	2 (0.2%)	0 (0%)	17 (1.8%)	14 (2.2%)	5 (1.5%)
Hispanic or latino ethnicity	138 (9.8%)	82 (9.8%)	6 (5.9%)	67 (7.2%)	50 (7.7%)	27 (8.1%)
Education						
No high school diploma	18 (1.3%)	11 (1.3%)	2 (2.0%)	15 (1.6%)	9 (1.4%)	6 (1.8%)
High school diploma or GED	201 (14.3%)	106 (12.7%)	18 (17.6%)	123 (13.1%)	79 (12.2%)	36 (10.8%)
Trade or vocational school	13 (0.9%)	8 (1.0%)	2 (2.0%)	34 (3.6%)	25 (3.9%)	13 (3.9%)
Some college but no degree	371 (26.4%)	230 (27.6%)	28 (27.5%)	217 (23.2%)	158 (24.4%)	93 (28%)
Associate Degree	93 (6.6%)	62 (7.4%)	11 (10.8%)	96 (10.3%)	72 (11.1%)	34 (10.2%)
Bachelor’s degree	453 (32.3%)	279 (33.5%)	33 (32.4%)	221 (23.6%)	137 (21.2%)	71 (21.4%)
Some graduate school but no degree	48 (3.4%)	23 (2.8%)	2 (2.0%)	53 (5.7%)	35 (5.4%)	19 (5.7%)
Master’s degree	160 (11.4%)	95 (11.4%)	6 (5.9%)	122 (13%)	93 (14.4%)	44 (13.3%)
Professional degree	25 (1.8%)	9 (1.1%)	0 (0%)	31 (3.3%)	21 (3.2%)	9 (2.7%)
Doctoral degree	21 (1.5%)	10 (1.2%)	0 (0%)	24 (2.6%)	18 (2.8%)	7 (2.1%)
DMQ-SS mean score (0–4)	0.70 (0.77)	0.99 (0.8)	2.28 (0.65)	1.36 (1.00)	1.67 (0.93)	2.19 (0.78)
DMQ *N*_*Triggers*_ (0–16)	2.77 (2.73)	3.92 (2.77)	6.91 (2.89)	4.97 (3.75)	6.28 (3.55)	7.66 (3.48)
DMQ impact VAS (0–100)	32.59 (24.53)	35.69 (24.69)	63.25 (19.21)	42.52 (29.37)	50.4 (26.82)	62.2 (23.22)
IHS total score (25–100)	40.88 (15.15)	45.19 (16.06)	70.33 (14.48)	59.86 (18.68)	65.53 (16.7)	75.87 (12.04)
IHS general loudness score (3–12)	4.94 (2.22)	5.51 (2.39)	8.74 (2.13)	2.76 (0.92)	3.04 (0.78)	3.45 (0.51)
DSM-SP phonophobia score (0–4)	0.45 (0.65)	0.59 (0.72)	1.65 (0.83)	1.16 (1.00)	1.38 (1.00)	1.89 (0.91)
OASIS total score (0–20)	5.88 (4.7)	6.68 (4.52)	10.94 (3.67)	−	−	−
ODSIS total score (0–20)	4.53 (5.07)	5.32 (5.2)	9.41 (5.58)	−	−	−
CUANGOS total score (0–20)	2.57 (3.28)	3.07 (3.44)	6.15 (4.58)	−	−	−
SSS-8 total score (0–32)	8.03 (5.97)	9.24 (6.08)	15.44 (6.32)	−	−	−
RLSS total score (6–42)	23.33 (8.85)	22.35 (8.82)	18.31 (7.22)	−	−	−

Continuous variables are presented as M (SD), whereas categorical variables are presented as N (%). S1 Positive, screen positive on DVMSQ “screening” item; DMQ-SS, Duke Misophonia Questionnaire–Symptom Scale; N_Triggers_, number of DMQ trigger categories endorsed; VAS, visual analog scale; IHS-LOUD, Inventory of Hyperacusis Symptoms “General Loudness” subscale; DSM-SP, DSM-5 Specific Phobia Severity Scale (modified for phonophobia); OASIS, Overall Anxiety Severity and Impairment Scale; ODSIS, Overall Depression Severity and Impairment Scale; CUANGOS, Clinically Useful Anger Outcome Scale; SSS-8, Somatic Symptom Scale–8; RLSS, Riverside Life Satisfaction Scale.

Based on the DVMSQ algorithm, a total of 102 individuals in the Prolific sample (7.3%, including 7 autistic adults) and 332 individuals in the SPARK sample (35.5%) met criteria for clinically significant misophonia. Subclinical misophonia, defined as meeting all DVMSQ criteria except for the “impairment” criterion, was present in an additional 144 adults in the Prolific sample (10.2%) and 97 adults in the SPARK sample (10.4%). Notably only 10 individuals in the Prolific sample (0.7%) and 21 individuals in the SPARK sample (2.2%) reported being previously diagnosed with misophonia by a professional, with almost all of these individuals meeting DVMSQ criteria for misophonia (Prolific: 7 Clinical, 2 Subclinical, 1 No misophonia; SPARK: 19 Clinical, 1 Subclinical, 1 No misophonia). Furthermore, over 85% of individuals with clinically significant misophonia in both samples (Prolific: 90.2%; SPARK: 85.5%) reported oronasal or throat sounds as among their misophonic triggers (defined as endorsing “*Mouth sounds while eating*,” “*Nasal/throat sounds*,” and/or “*Mouth sounds while not eating*” on the DMQ trigger list).

Within the Prolific sample, individuals meeting criteria for clinically significant misophonia were slightly younger (Misophonia: 29.69 years, Other: 32.48 years; *d* = −0.22, CI_95%_ [−0.42, −0.02]), more likely to be female (Misophonia: 72.5%, Other: 49.4%; OR = 2.70, CI_95%_ [1.73, 4.23]), and less likely to have completed a 4-year college degree (Misophonia: 40.2%, Other: 51.2%; OR = 0.64, CI_95%_ [0.43, 0.97]) than individuals with no clinically significant misophonia. Although the association between misophonia and female sex was similarly robust in the SPARK sample (OR = 3.07, CI_95%_ [2.26, 4.18]), associations with younger age (*d* = −0.04, CI_95%_ [−0.17, 0.10]) and lower college completion (OR = 0.83, CI_95%_ [0.63, 1.09]) were much smaller and not statistically significant. Additionally, as expected, individuals meeting DVMSQ misophonia criteria demonstrated much higher scores on the DMQ-SS (Prolific: *d* = 2.71, CI_95%_ [2.49, 2.94]; SPARK: *d* = 1.65, CI_95%_ [1.50, 1.80]), more reported misophonia triggers (Prolific: *d* = 1.80, CI_95%_ [1.59, 2.01]; SPARK: *d* = 1.31, CI_95%_ [1.17, 1.46]), and higher VAS scores for misophonia-related impairment (Prolific: *d* = 1.52, CI_95%_ [1.30, 1.73]; SPARK: *d* = 1.20, CI_95%_ [1.05, 1.34]) than individuals without DVMSQ-defined misophonia. For individuals in the Prolific sample, misophonia status was also strongly associated with higher levels of anxiety (OASIS; *d* = 1.22, CI_95%_ [1.01, 1.42]), depression (ODSIS; *d* = 1.08, CI_95%_ [0.87, 1.28]), anger (CUANGOS; *d* = 1.24, CI_95%_ [1.03, 1.44]), and somatic symptom burden (SSS-8; *d* = 1.43, CI_95%_ [1.22, 1.64]), as well as lower reported quality of life (RLSS; *d* = −0.62, CI_95%_ [−0.82, −0.42]).

### Item analysis

Duke-vanderbilt misophonia Screening Questionnaire item category frequencies, percentages of each item fulfilling its associated operational diagnostic criterion, item-total correlations, and correlations with other sound tolerance measures (DMQ-SS, IHS-LOUD, and DSM-SP) are presented for the Prolific sample in Table 4. Item endorsement at levels corresponding to the diagnostic criteria was highly variable and ranged from 7.4% (*Impairment – Community*) to 55.2% (*Intense irritation or annoyance*). Corrected item-total polyserial correlations were high (median *r*_*it*_ = 0.677, IQR [0.636, 0.765]), with all correlations greater than 0.5 with the exception of item 4 (*Disgust*). Polyserial correlations between DVMSQ items and the DMQ-SS (median *r*_*poly*_ = 0.601, IQR [0.565, 0.639]) were somewhat higher on average than correlations with either the IHS-LOUD subscale (median *r*_*poly*_ = 0.479, IQR [0.405, 0.634]) or the DSM-SP score (median *r*_*poly*_ = 0.479, IQR [0.417, 0.619]). Notably, the DVMSQ items assessing misophonia *symptoms* tended to correlate more strongly with the DMQ-SS than the IHS-LOUD or DSM-SP, but this was not typically the case for the DVMSQ *impairment* items, several of which correlated more strongly with the IHS-LOUD and/or DSM-SP than the DMQ-SS.

**TABLE 4:** Item characteristics in Prolific sample that screened positive on DVMSQ item S1 (*n* = 833).

Item	Abbreviated content	Response distribution (0/1/2/3/4)	Fulfills criterion	*r*_*it*_	*r*_*DMQ–SS*_	*r*_*DSM–SP*_	*r*_*IHS*–LOUD_
1	Intense irritation or annoyance	17/72/284/296/164	55.2%	0.610	0.558	0.312	0.321
2	Anger or rage	189/229/227/127/61	22.6%	0.650	0.638	0.337	0.328
3	Fear or panic	514/175/88/39/17	−	0.639	0.575	0.553	0.499
4	Disgust	200/137/247/165/84	29.9%	0.380	0.383	0.216	0.164
5	Urge to run away from sound	259/173/188/143/70	25.6%	0.682	0.582	0.424	0.380
6	Urge to block out sound	135/160/241/161/136	35.7%	0.628	0.565	0.427	0.413
7	Violent urges	454/182/109/56/32	23.6%	0.649	0.594	0.305	0.317
8	Loss of control	353/163/164/103/50	38.1%	0.718	0.608	0.403	0.430
9	Attention capture by trigger	87/131/250/217/148	−	0.696	0.623	0.437	0.460
10	Physical response	405/103/146/103/76	−	0.598	0.554	0.471	0.435
11	Excessive/unreasonable reactions	156/272/239/121/45	−	0.665	0.626	0.421	0.504
12	Avoidance of triggers	218/219/226/122/48	47.5%	0.595	0.489	0.488	0.459
13	Impairment – Social	553/190/63/19/8	10.8%	0.837	0.654	0.624	0.670
14	Impairment – Occupational	513/192/86/32/10	15.4%	0.672	0.564	0.539	0.536
15	Impairment – Domestic	640/115/55/14/9	9.4%	0.803	0.619	0.623	0.634
16	Impairment – Community	685/86/38/17/7	7.4%	0.752	0.572	0.618	0.636
17	Impairment – Concentration	233/289/171/103/37	−	0.690	0.644	0.598	0.607
18	Global impact – Mental health	430/240/104/41/17	19.5%	0.838	0.695	0.665	0.723
19	Global impact – Created problems	430/252/89/44/18	18.1%	0.834	0.697	0.675	0.726
20	Global impact – Life worse	595/151/47/24/15	10.4%	0.867	0.660	0.634	0.704

“Fulfills criterion” indicates the percentage of the 833 respondents whose response to a given item was sufficient to fulfill a given DVMSQ-based criterion from Table 2 (i.e., a score of ≥2 or ≥3 depending on the specific item; see Table 2). r_it_, corrected polyserial item-total correlation; r_DMQ–SS_, polyserial correlation between item and Duke Misophonia Questionnaire–Symptom Scale total score; r_DSM–SP_, polyserial correlation between item and DSM-5 Specific Phobia Severity Scale (modified for phonophobia); r_IHS–LOUD_, polyserial correlation between item and Inventory of Hyperacusis Symptoms “General Loudness” subscale.

### Dimensionality assessment

Exploratory graph analysis of the original 20 DVMSQ items was conducted in the subset of Prolific participants who screened positive on DVMSQ item S1 (*n* = 833), revealing a partial correlation network with four communities. These communities were interpreted as *Symptoms: Anger and Aggression* (items 1, 2, 4, and 7). *Symptoms: Distress and Avoidance* (items 5, 6, 8, 9, 10, 11, and 12); *Impairment: Specific* (items 13, 14, 15, 16, and 17); and *Impairment: Global Impact* (items 3, 18, 19, and 20). Notably, item 3 (*Fear or panic*) was assigned to the “Impairment: Global Impact” community rather than either of the symptom communities, suggesting that it likely represented a separate latent variable than the other items tapping distress and avoidance. Thus, item 3 was dropped from the model, and the EGA was repeated with the remaining items. After removing item 3, the dimensionality and community structure of the 19 remaining items did not change, and this structure was then used to inform the structure of IRT models for both symptoms (11 items) and impairment (8 items). Further converging with the results of the EGA, the Factor Forest method also found a four-dimensional structure to be most likely, both before (*P*_*k* = 4_ = 0.865) and after (*P*_*k* = 4_ = 0.801) removing item 3.

### Item response theory analyses

#### Misophonia symptoms

In the exploratory subsample of Prolific participants, we first fit the 11 symptom items with a bifactor model, in which all items loaded on one general factor, and each item additionally loaded on a specific factor based on its community assignment within the EGA (i.e., items 1, 2, 4, and 7 on specific factor 1 and items 5, 6, 8, 9, 10, 11, and 12 on specific factor 2). This initial model demonstrated global fit indices that were adequate overall (*C*_2_(33) = 92.55, *p* < 0.001, TLI_*C*2_ = 0.976, RMSEA_*C*2_ = 0.066, CI_90%_ [0.050, 0.082], SRMR = 0.041), and no local dependence, but two standardized residuals (items 4 [*Disgust*] and 5 [*Urge to run away*]: *r*_*res*_ = 0.156; items 4 [*Disgust*] and 11 [*Excessive/unreasonable reactions*]: *r*_*res*_ = −0.115) were greater than 0.1, suggesting additional local misspecification. Furthermore, while examination of factor loadings in this model demonstrated large positive loadings on the general factor for all items (median λ_*G*_ = 0.714, range [0.464, 0.815]), loadings on the “Distress and Avoidance” factor were negligible for items 8 (*Loss of control*; λ_*S*2_ = −0.065) and 9 (*Attention capture by trigger*; λ_*S*2_ = 0.008). Item 11 also demonstrated an unexpected loading pattern, with a strong general factor loading (λ_*G*_ = 0.743) and a moderate negative loading on the “Distress and Avoidance” factor (λ_*S*2_ = −0.309). Thus, to correct the model misspecification, we allowed item 4 to load onto both specific factors, fixed the loadings of items 8 and 9 on specific factor 2 to 0, and removed item 11 (*Excessive/unreasonable reactions*) from the model entirely. The resulting model demonstrated significantly improved fit in the exploratory subsample (*C*_2_(26) = 33.53, *p* = 0.147, TLI_*C*2_ = 0.996, RMSEA_*C*2_ = 0.026, CI_90%_ [0.000, 0.050], SRMR = 0.028), no local dependence, no large residuals, and factor loadings all greater than 0.1. This same model was then re-fit in the confirmatory subsample, again demonstrating adequate fit (*C*_2_(26) = 67.19, *p* < 0.001, TLI_*C*2_ = 0.979, RMSEA_*C*2_ = 0.062, CI_90%_ [0.044, 0.080], SRMR = 0.037) and no local dependence. One residual correlation between items 7 (*Violent urges*) and 8 (*Loss of control*) was above the cutoff value in the confirmatory sample (*r*_*res*_ = 0.112); however, allowing item 8 to load onto the “Anger and Aggression” factor produced a standardized loading of <0.1; thus, the model without this loading was retained as our final symptom model. IRT model parameters, factor loadings and bifactor coefficients for the final symptom model (fit to the combined exploratory and confirmatory Prolific samples) are presented in Supplementary Tables 1, 2. Bifactor coefficients indicated high reliability (ω_*T*_ = 0.927), with the majority of variance accounted for by a single general “misophonia symptoms” factor (ω_*H*_ = 0.825, ECV = 0.747).

#### Misophonia-related impairment

In the exploratory subsample of Prolific participants, we fit the eight impairment items with a bifactor S–1 model (Eid et al., 2018), in which all items loaded onto a single general factor and the three “Global Impact” items loaded onto a single specific factor in accordance with their EGA community assignment. This initial model demonstrated adequate global fit (*C*_2_(17) = 55.19, *p* < 0.001, TLI_*C*2_ = 0.985, RMSEA_*C*2_ = 0.073, CI_90%_ [0.052, 0.095], SRMR = 0.041), no local dependence, and no large residuals. Additionally, loadings on the general factor were strong for all items (median λ_*G*_ = 0.846, range [0.817, 0.886]), and all specific factor loadings were greater than 0.1. However, when this model was re-fit in the confirmatory subsample, the global fit was substantially worse (*C*_2_(17) = 75.01, *p* < 0.001, TLI_*C*2_ = 0.978, RMSEA_*C*2_ = 0.091, CI_90%_ [0.070, 0.112], SRMR = 0.053). This decrement in fit was accompanied by two large residual pairs (item 14 [*Impairment – Occupational*] and item 17 [*Impairment – Concentration*]: *r*_*res*_ = 0.122; item 14 and item 20 [*Global impact – Life worse*]: *r*_*res*_ = −0.120), as well as significant local dependence between items 14 and 17 (Q_3_ = 0.276 [99th percentile: 0.233]). In response to this local misfit, we removed item 17 from the model, resulting in adequate global fit (*C*_2_(11) = 19.95, *p* = 0.046, TLI_*C*2_ = 0.995, RMSEA_*C*2_ = 0.044, CI_90%_ [0.006, 0.075], SRMR = 0.035), no large residuals, and no locally dependent item pairs within the confirmatory sample. This model also fit well within the exploratory sample *C*_2_(11) = 18.89, *p* = 0.063, TLI_*C*2_ = 0.995, RMSEA_*C*2_ = 0.042, CI_90%_ [0.000, 0.072], SRMR = 0.033, again demonstrating no local misfit or local item dependence. Thus, it was retained as the final model. IRT model parameters, factor loadings and bifactor coefficients for the final impairment model (fit to the combined exploratory and confirmatory Prolific samples) are presented in Supplementary Tables 3, 4. Bifactor coefficients indicated high reliability (ω_*T*_ = 0.958), with almost all reliable variance accounted for by a single general “misophonia-related impairment” factor (ω_*H*_ = 0.905, ECV = 0.902).

#### All duke-vanderbilt misophonia screening questionnaire items (symptoms and impairment)

Within the exploratory Prolific subsample, we fit a bifactor model to all 17 remaining DVMSQ items, including one general factor and four specific factors (*Anger and Aggression*: items 1, 2, 4, and 7; *Distress and Avoidance*: items 4, 5, 6, 10, and 12; *Overall Impairment*: items 13, 14, 15, 16, 18, 19, and 20; and *Global Impact*: items 18, 19, and 20). This model fit the data in the exploratory subsample well (*C*_2_(100) = 144.33, *p* = 0.002, TLI_*C*2_ = 0.994, RMSEA_*C*2_ = 0.033, CI_90%_ [0.020, 0.044], SRMR = 0.044) and demonstrated no local dependence. However, two residual correlations exceeded 0.15 (item 12 [*Avoidance of triggers*] and item 13 [*Impairment – Social*]: *r*_*res*_ = 0.163; item 12 and item 16 [*Impairment – Community*]: *r*_*res*_ = 0.152), prompting us to allow item 12 to cross-load onto the “Overall Impairment” factor. The revised model demonstrated slightly improved fit (*C*_2_(99) = 129.62, *p* = 0.021, TLI_*C*2_ = 0.996, RMSEA_*C*2_ = 0.027, CI_90%_ [0.011, 0.039], SRMR = 0.035), no residual correlations greater than 0.15, and no locally dependent item pairs. This revised model was then re-fit in the confirmatory sample, again exhibiting adequate global fit (*C*_2_(99) = 188.05, *p* < 0.001, TLI_*C*2_ = 0.988, RMSEA_*C*2_ = 0.047, CI_90%_ [0.036, 0.057], SRMR = 0.046), no large residuals, and no local dependence. The same model fit in the SPARK sample also demonstrated adequate fit in the population of autistic adults (*C*_2_(99) = 196.06, *p* < 0.001, TLI_*C*2_ = 0.991, RMSEA_*C*2_ = 0.039, CI_90%_ [0.031, 0.047], SRMR = 0.042), as well as no local misfit or locally dependent item pairs. Parameters for the combined model in both the Prolific and SPARK samples are presented in Table 5. Notably, while reliability of the DVMSQ total score was very high in both samples (Prolific: ω_*T*_ = 0.977; SPARK: ω_*T*_ = 0.957; Supplementary Tables 5, 6), the “general misophonia” factor explained a smaller relative proportion of total score variance (Prolific: ω_*H*_ = 0.756, ECV = 0.586; SPARK: ω_*H*_ = 0.740, ECV = 0.567), seemingly due to the sizable minority of variance in DVMSQ impairment items attributed to the “Overall Impairment” factor (Prolific: ω_*HS*_ = 0.457; SPARK: ω_*HS*_ = 0.474). Moreover, the DVMSQ symptom and impairment scales demonstrated significant convergence (Prolific: *r* = 0.560, CI_95%_ [0.512, 0.605]; SPARK: *r* = 0.617, CI_95%_ [0.567, 0.662]), although the magnitude of their intercorrelation was low enough to suggest that the two scores may differentially correlate with external variables in some cases (Carlson and Herdman, 2012).

**TABLE 5:** Bifactor graded response model parameters for the final DVMSQ model in Prolific and SPARK samples.

	Prolific sample										Spark sample								
	*a*_1_	*a*_2_	*a*_3_	*a*_4_	*a*_5_	*d*_1_	*d*_2_	*d*_3_	*d*_4_	*a*_1_		*a*_2_	*a*_3_	*a*_4_	*a*_5_	*d*_1_	*d*_2_	*d*_3_	*d*_4_
Item 1	1.96	1.09	−	−	−	5.69	3.46	0.49	−2.35	1.60		0.77	−	−	−	6.03	4.48	2.06	−0.01
Item 2	5.15	5.83	−	−	−	6.03	0.41	−5.70	−11.90	4.32		5.36	−	−	−	7.38	3.59	−2.27	−6.93
Item 4	0.82	0.55	0.66	−	−	1.46	0.56	−0.98	−2.66	1.00		0.64	−0.07	−	−	1.82	0.78	−0.53	−1.97
Item 5	2.85	−	2.17	−	−	2.09	0.07	−2.44	−5.61	2.34		−	1.00	−	−	4.06	2.80	1.10	−0.90
Item 6	2.07	−	0.97	−	−	2.80	1.16	−0.92	−2.76	2.41		−	1.84	−	−	6.74	5.23	2.64	0.45
Item 7	1.78	1.37	−	−	−	−0.16	−1.85	−3.48	−5.18	1.55		1.40	−	−	−	1.16	−0.29	−2.01	−3.39
Item 8	2.24	−	−	−	−	0.65	−0.68	−2.43	−4.55	1.70		−	−	−	−	2.61	1.29	−0.29	−1.73
Item 9	2.47	−	−	−	−	3.75	1.98	−0.34	−2.75	2.27		−	−	−	−	6.25	4.59	1.91	0.03
Item 10	1.32	−	0.30	−	−	0.18	−0.47	−1.60	−2.91	1.31		−	0.11	−	−	1.73	1.34	0.28	−0.94
Item 12	1.29	−	0.30	−	−	1.39	−0.10	−1.75	−3.49	1.25		−	0.25	0.49	−	3.54	2.21	0.41	−1.20
Item 13	2.53	−	−	2.59	−	−1.67	−4.96	−7.52	−9.60	2.44		−	−	3.25	−	2.68	−0.80	−4.67	−7.70
Item 14	1.38	−	−	1.70	−	−0.79	−2.80	−4.61	−6.52	1.43		−	−	2.42	−	0.91	−1.00	−3.13	−5.14
Item 15	2.12	−	−	2.67	−	−2.75	−4.92	−7.26	−8.83	1.69		−	−	2.25	−	0.75	−1.39	−3.67	−6.04
Item 16	1.79	−	−	2.73	−	−3.36	−5.24	−6.92	−8.87	1.25		−	−	1.98	−	0.04	−0.98	−2.40	−3.73
Item 18	3.52	−	−	2.87	1.98	−0.13	−4.35	−7.66	−10.69	3.00		−	−	2.55	1.99	5.51	1.26	−2.82	−6.30
Item 19	5.33	−	−	4.28	3.52	−0.18	−6.89	−11.19	−15.65	3.57		−	−	2.82	2.74	7.31	1.83	−2.52	−7.34
Item 20	3.35	−	−	2.78	1.84	−2.67	−6.17	−8.19	−10.46	2.56		−	−	1.90	1.83	2.85	−0.30	−3.43	−5.77

Both models assume that all latent variables are orthogonal and have a standard normal distribution (i.e., M = 0, SD = 1) in the population. Differences in intercept terms between the two groups are not significant after considering the higher mean scores on the misophonia latent trait in the SPARK sample. a_1_–a_5_ = slope parameters (higher values indicate stronger relationships with the latent variables [i.e., stronger factor loadings]); d_1_–d_4_ = intercept parameters (higher values indicate “less difficult” or more easily endorsed item categories).

For the 17 DVMSQ items included in the final model, DIF was evaluated using the iterative Wald test procedure. Based on these tests, statistically significant DIF (i.e., *p*_*FDR*_ < 0.05 and | ESSD| > 0.2) was detected in four DVMSQ items (4, 10, 12, and 16; Table 6), although only the DIF in item 12 (*Avoidance of triggers*; ESSD = 0.587) was large enough to meet our threshold of practical significance. Moreover, the total impact of DIF on between-group score differences was relatively low, on average summing to a less-than-one point difference on a 68-point scale (UETSDS = 0.722, ETSSD = 0.053). As this was within the amount of DTF that we deemed ignorable in practice (i.e., | ETSSD| < 0.1), we concluded that the DVMSQ is approximately invariant according to autism status. DIF was also evaluated within the Prolific sample with respect to age group (<30 years old vs. ≥30 years old), sex (female vs. male), and education level (any college degree vs. “some college” or less). No statistically significant DIF was found according to age (all *p*_*FDR*_ > 0.169; all | ESSD| < 0.387) or education level (all *p*_*FDR*_ > 0.607; all | ESSD| < 0.192), although practically significant DIF between males and females was observed for item 12 (ESSD = 0.650). However, as in the case of DIF by diagnostic group, the degree of DTF by sex was less than one DVMSQ scale point (UETSDS = 0.420, ETSSD = 0.040) and was deemed small enough to not result in a practically significant amount of bias. Thus, based on these results, the DVMSQ was judged to be approximately invariant across age, sex, education level, and diagnostic status.

**TABLE 6:** Differential item functioning test results for non-invariant items.

Item	Grouping variable	χ^2^	*df*	*p*_*FDR*_	UIDS	ESSD	Non-invariant parameters
4	Autism diagnosis	29.65	7	<0.001	0.290	−0.398	Slopes (*a*_1_ higher in AUT, *a*_3_ lower in AUT) Intercepts (all lower in AUT)
10	Autism diagnosis	19.90	6	0.003	0.211	0.260	Intercepts (*d*_1_ lower in AUT, *d*_2–4_ higher in AUT)
**12**	**Autism diagnosis**	**37.63**	**6**	**<0.001**	**0.379**	**0.587**	Intercepts (all higher in AUT)
16	Autism diagnosis	32.35	6	<0.001	0.358	0.374	Intercepts (all higher in AUT)
**12**	**Sex**	**34.64**	**6**	**<0.001**	**0.420**	**0.650**	Intercepts (all higher in Males)

Results indicate omnibus Wald tests of differential item functioning (DIF) using a version of the iterative anchor-selection method of Cao et al. (2017). Items presented in bold demonstrated differential item functioning large enough to be deemed “practically significant” (i.e., |ESSD| > 0.5). Parameter groups (i.e., either slopes or intercepts) that were significantly different between groups when tested alone with follow-up Wald tests (p < 0.05, uncorrected) are indicated in the “Non-invariant Parameters” column. Higher intercepts indicate less item difficulty (i.e., more item endorsement at a given latent trait level). UIDS, unsigned item difference in the sample (unsigned DIF effect size in response scale units); ESSD, expected score standardized difference (signed DIF effect size in Cohen’s d units); AUT, autism group.

### Validity testing

Zero-order correlations between the DVMSQ scales and external variables of interest are presented in Table 7. Notably, the DVMSQ total score correlated very highly with the DMQ-SS score in both the full sample (*r* = 0.802, CI_95%_ [0.783, 0.820]) and the S1 Positive sample (*r* = 0.855, CI_95%_ [0.835, 0.872]), strongly supporting the convergent validity of these two measures. Although most observed correlations were similar in magnitude to our predictions, correlations between all DVMSQ scores and non-misophonia forms of decreased sound tolerance (i.e., the IHS-LOUD and DSM-SP) were substantially larger than expected, and correlations between the DVMSQ scores and the RLSS were somewhat smaller than expected. When statistically comparing correlation coefficients, the DVMSQ total score was more strongly correlated with the DMQ-SS than either the IHS-LOUD score (Whole Sample: Δ*r* = 0.192, CI_95%_ [0.165, 0.222]; DVMSQ-complete Sample: Δ*r* = 0.175, CI_95%_ [0.143, 0.210]) or the DSM-SP score (Whole Sample: Δ*r* = 0.204, CI_95%_ [0.177, 0.233]; DVMSQ-complete Sample: Δ*r* = 0.153, CI_95%_ [0.124, 0.185]), providing modest evidence of divergent validity despite the relatively high correlations with measures of hyperacusis and misophonia. This same pattern of correlation differences was present for the DVMSQ symptom score but not the DVMSQ impairment score (Table 7), suggesting that the latter score does not necessarily differentiate impairment due to misophonia from impairment due to other forms of decreased sound tolerance. Lastly, contrary to our hypotheses, correlations between the DVMSQ and the CUANGOS were not uniformly larger than correlations between the DVMSQ and the OASIS, ODSIS, or SSS-8 (Whole Sample: Δ*r*s = −0.048–0.038; DVMSQ-complete Sample: Δ*r*s = −0.021–0.063), with similar patterns observed for both the DVMSQ symptom and impairment subscales as well. In fact, in all but one case (symptom score in the DVMSQ-complete Sample), somatic symptom burden was a stronger correlate of the DVMSQ than depression, anxiety, or anger, although absolute differences between correlations were generally small in magnitude (all Δ*r*s < 0.1).

**TABLE 7:** Pearson correlations between DVMSQ scores and external variables.

	DVMSQ correlations (Full sample)			DVMSQ correlations (S1 positive sample)		
	Total score	Symptom score	Impairment score	Total score	Symptom score	Impairment score
DMQ-SS (misophonia)	0.802 [0.783, 0.820]	0.751 [0.727, 0.773]	0.723 [0.697, 0.747]	0.855 [0.835, 0.872]	0.782 [0.754, 0.807]	0.728 [0.694, 0.758]
DMQ *N*_*Triggers*_	0.641 [0.610, 0.671]	0.598 [0.563, 0.630]	0.584 [0.549, 0.618]	0.507 [0.455, 0.556]	0.413 [0.355, 0.468]	0.518 [0.467, 0.566]
DMQ Frequency	0.556 [0.519, 0.591]	0.493 [0.453, 0.532]	0.564 [0.527, 0.598]	0.600 [0.555, 0.642]	0.499 [0.446, 0.548]	0.595 [0.549, 0.637]
DMQ Impairment VAS	0.506 [0.466, 0.544]	0.429 [0.386, 0.471]	0.558 [0.521, 0.593]	0.605 [0.560, 0.646]	0.494 [0.441, 0.544]	0.614 [0.570, 0.655]
IHS-LOUD (hyperacusis)	0.609 [0.575, 0.641]	0.509 [0.469, 0.547]	0.699 [0.671, 0.725]	0.680 [0.641, 0.714]	0.517 [0.465, 0.565]	0.757 [0.727, 0.785]
DSM-SP (phonophobia)	0.599 [0.564, 0.631]	0.491 [0.450, 0.530]	0.709 [0.682, 0.734]	0.701 [0.665, 0.734]	0.537 [0.487, 0.584]	0.775 [0.746, 0.800]
OASIS (anxiety)	0.383 [0.338, 0.427]	0.330 [0.283, 0.376]	0.414 [0.370, 0.457]	0.459 [0.404, 0.511]	0.364 [0.304, 0.422]	0.486 [0.433, 0.536]
ODSIS (depression)	0.345 [0.298, 0.390]	0.300 [0.252, 0.347]	0.366 [0.320, 0.411]	0.385 [0.326, 0.442]	0.311 [0.248, 0.371]	0.400 [0.341, 0.455]
CUANGOS (anger)	0.383 [0.337, 0.427]	0.338 [0.291, 0.384]	0.394 [0.349, 0.437]	0.448 [0.392, 0.501]	0.381 [0.322, 0.438]	0.430 [0.373, 0.484]
SSS-8 (somatic symptoms)	0.431 [0.388, 0.473]	0.376 [0.330, 0.420]	0.456 [0.414, 0.497]	0.469 [0.414, 0.520]	0.374 [0.314, 0.431]	0.494 [0.441, 0.544]
RLSS (quality of life)	−0.225 [−0.274, −0.175]	−0.201 [−0.251, −0.151]	−0.226 [−0.275, −0.176]	−0.243 [−0.306, −0.178]	−0.201 [−0.266, −0.135]	−0.243 [−0.306, −0.178]

All correlations are presented with their 95% confidence intervals. “Full Sample” refers to the full Prolific sample (n = 1403), whereas “DVMSQ-complete Sample” refers to the subset of individuals who answered “Yes” to the duke-vanderbilt Misophonia Screening Questionnaire (DVMSQ) screening question and completed all additional DVMSQ questions (n = 833). DMQ-SS, Duke Misophonia Questionnaire–Symptom Scale; N_Triggers_, number of DMQ trigger categories endorsed; VAS, visual analog scale; IHS-LOUD, Inventory of Hyperacusis Symptoms “General Loudness” subscale; DSM-SP, DSM-5 Specific Phobia Severity Scale (modified for phonophobia); OASIS, Overall Anxiety Severity and Impairment Scale; ODSIS, Overall Depression Severity and Impairment Scale; CUANGOS, Clinically Useful Anger Outcome Scale; SSS-8, Somatic Symptom Scale–8; RLSS, Riverside Life Satisfaction Scale.

## Discussion

Though a number of novel self-report questionnaires have been published in the past several years to assess the symptoms of misophonia, there is still limited consensus regarding the most suitable measures for different purposes within misophonia research and clinical care (e.g., diagnosis, screening, clinical phenotyping, longitudinal symptom tracking, quantifying response to intervention). In the current study, we introduced and examined the psychometric properties of the DVMSQ, a brief measure of misophonia symptoms and associated impairment designed specifically to assess a set of operational diagnostic criteria and determine “misophonia caseness” in the context of research studies. Examining DVMSQ responses from over 2,000 autistic and non-autistic adults, we iteratively tested and subsequently replicated the latent structure of the questionnaire, which was found to be approximately invariant according to autism status, age group, sex, and level of education. Model-based reliability of the DVMSQ total score was high, and the pattern of correlations between the DVMSQ and other related variables strongly supported its construct validity as a measure of misophonia severity and impairment. Although further studies are needed to establish the diagnostic efficiency (e.g., sensitivity, specificity, and positive/negative predictive values), temporal stability, and sensitivity-to-change of this measure, initial psychometric data on the DVMSQ support its use as a measure of misophonia symptoms and impairment in both general population adults and adults on the autism spectrum. The revised DVMSQ form is freely available for use and can be found in Supplementary material.

By incorporating the recent consensus definition of misophonia (Swedo et al., 2022) into our DVMSQ-based diagnostic algorithm, this study represents the first attempt to operationalize the misophonia consensus definition into a formal set of diagnostic criteria to be applied in research or clinical practice (Williams, 2022). Using the DVMSQ algorithm to define misophonia caseness, the prevalence of clinically significant misophonia was 7.3% (102/1403) in a sex-balanced crowdsourced sample from Prolific and 35.5% (332/936) in a female-predominant sample of independent autistic adults recruited from the SPARK cohort. An additional 10% of each sample (i.e., 144 adults in the Prolific sample and 97 adults in the SPARK sample) met DVMSQ criteria for “subclinical misophonia” (i.e., misophonia symptoms above the clinical threshold but without significant functional impairment). Notably, these prevalence figures may be modestly overestimated due to the selection bias of individuals with misophonia preferentially participating in our studies, which were advertised as being about “sensory sensitivities,” broadly defined. Misophonia status in both general population and autistic samples was linked to female sex, and in the general population only, younger age and lower college completion rates. Though the DVMSQ-derived categories of clinical and subclinical misophonia have yet to be validated using independent criteria (e.g., best-estimate clinical diagnosis of misophonia based on a structured interview), the general-population estimates observed here are similar to those derived in the only interview-based epidemiological study of misophonia prevalence conducted to date (Kılıç et al., 2021). Moreover, 29 of 31 individuals in the current study who had previously received clinical diagnoses of misophonia (93.5%) were flagged by the DVMSQ as meeting all misophonia symptom criteria necessary for a diagnosis, providing further evidence to support the validity of the screening algorithm.

This study was also the first to examine the prevalence and features of misophonia in a sample of autistic adults, a clinical population that anecdotally reports high rates of misophonia-like symptoms (Landon et al., 2016; Scheerer et al., 2021; Williams et al., 2021c) but that has not previously been systematically studied using validated misophonia symptom measures. Based on the DVMSQ criteria, clinically significant misophonia was present in slightly over one-third of our SPARK sample (44.2% of autistic females and 20.5% of autistic males), a rate substantially higher than that found in the general population Prolific sample. Notably, autistic individuals have been largely excluded from misophonia research to date (though see Haq et al., 2021; Tonarely-Busto et al., 2022), potentially due to prior iterations of misophonia diagnostic criteria attempting to differentiate misophonia from other forms of decreased sound tolerance often observed in autism (Schröder et al., 2013; Jager et al., 2020). Though a substantial majority of individuals with clinically significant misophonia are likely non-autistic (e.g., only 7 of 102 in our Prolific sample [6.9%] reported a formal autism diagnosis), our data demonstrate that many autistic individuals meet full criteria for misophonia, and that most of these individuals (around 85%) report “classic” oronasal sounds as among their specific triggers. Furthermore, empirical analyses of the DVMSQ found that the structure of misophonia symptoms does not differ meaningfully between autistic and non-autistic adults, with practically ignorable amounts of DTF between groups. These data suggest that misophonia associated with autism is not a qualitatively different entity from misophonia in non-autistic individuals, providing empirical support for the idea that misophonia should be considered a separate diagnostic entity in autistic individuals rather than being attributed to autism-associated sensory reactivity (Swedo et al., 2022). Though misophonia is likely less prevalent than hyperacusis in autistic individuals (Williams et al., 2021e; Carson et al., 2022), both disorders appear to contribute substantially to the overall burden of decreased sound tolerance in the autistic population, arguably warranting additional attention within autism research and specialist autism clinics. As the DVMSQ is the first measure of misophonia symptoms and impairment validated for use in the autistic population, autism researchers and clinicians treating autistic adults may find this measure particularly useful for understanding the misophonia phenotype in autism and monitoring the success of treatments aimed at reducing misophonia symptoms.

When examining the latent structure of the DVMSQ, we found that the scale’s items conformed to a bifactor structure with specific dimensions of anger/aggression, distress/avoidance, impairment, and global impact. Notably, the final measurement model excluded three of the original 20 DVMSQ items, namely (a) panic or fear in response to trigger stimuli, (b) perceptions of one’s misophonic reactions as being excessive or unreasonable, and (c) impairment in one’s ability to concentrate. With regard to the fear/panic item, it is notable that this item was endorsed at substantially lower rates than other emotional responses thought to be more typical of misophonia (i.e., irritation/annoyance, anger, and disgust). Furthermore, the fear/panic item was not assigned to either symptom-related community in the EGA, suggesting that it represented a latent construct separate from anger/aggression and distress/avoidance. This finding is in concordance with the large study of Jager et al. (2020), which found that despite individuals with misophonia reporting anticipatory anxiety surrounding triggers, none reported that the triggers themselves evoked feelings of fear or anxiety in the same manner that they evoked anger and/or disgust. Although the item assessing fear/panic was removed from the DVMSQ total score, we chose to retain it in the revised DVMSQ questionnaire in order to capture information about these emotions that may be relevant in deciding whether an individual has misophonia, phonophobia, or a combination thereof. The other two items excluded from the measurement model were both removed from the questionnaire, as neither was judged to contribute meaningful diagnostic information on its own in the way that the fear/panic item does. Since the collection of the data in the current study, the text of the initial DVMSQ “screening” item has also been modified to contain the following clarifying text: “*These sounds should cause significant emotional distress (e.g., extreme irritation, anger, disgust, rage, anxiety, or panic). Do NOT count sounds that bother you only because you find them too loud or physically painful.*” Though this version of the DVMSQ screening question has not been empirically tested, we believe that this clarifying text will be helpful in increasing the measure’s specificity for misophonia (i.e., eliminating false-positive “Yes” responses due to hyperacusis) without lowering its sensitivity for persons with misophonia who would have otherwise responded affirmatively to that initial question. Full text of the updated DVMSQ and scoring guidelines can be found in the Supplementary material.

The present study also investigated the construct validity of the DVMSQ and its component scores by examining correlations between these measures and (a) another psychometrically validated misophonia questionnaire (the DMQ), (b) measures of other forms of decreased sound tolerance (the IHS-LOUD and DSM-SP, measuring hyperacusis and phonophobia, respectively), (c) measures of psychopathology and somatic symptom burden (the OASIS, ODSIS, CUANGOS, and SSS-8), and a measure of general life satisfaction (the RLSS). Correlations between the DMVSQ and the DMQ-SS were exceptionally high (*r*s > 0.8 for the DVMSQ total score and *r*s > 0.7 for the symptom/impairment scores), supporting the convergent validity of these two misophonia severity measures in the general population (Carlson and Herdman, 2012). Correlations with other DMQ-derived measures, including the number of trigger categories, the frequency of trigger exposure, and the impact on one’s life, were lower but still in the moderate-to-large (0.4–0.65) range, suggesting that these aspects of misophonia are separable but related constructs. The DVMSQ also correlated with measures of anxiety, depression, anger, and somatic symptom burden in a way similar to our hypotheses. However, contrary to our predictions, misophonia symptoms did not correlate more strongly with anger than with other forms of emotional distress or somatic symptoms. Correlations between the DVMSQ scores and quality of life were also slightly smaller than predicted, although all were non-zero and in the anticipated direction.

Notably, discriminant correlations between the DVMSQ and other measures of non-misophonia decreased sound tolerance (i.e., the IHS-LOUD and the DSM-SP) were unexpectedly high, particularly for the DVMSQ impairment score. Though the DVMSQ total and symptom scores demonstrated significantly stronger correlations with measures of misophonia symptoms as opposed to hyperacusis/phonophobia symptoms, this was not the case for the DVMSQ impairment score, which shared similar amounts of variance with the measures of misophonia, hyperacusis, and phonophobia symptoms. This finding suggests that the “misophonia-related impairment” domain of the DVMSQ likely measures more general impairment due to all types of decreased sound tolerance. Although the differential correlations between measures of decreased sound tolerance do provide some evidence of discriminant validity for the DVMSQ total and symptom scores, measures of “misophonia symptoms” in the general population may potentially be substantially confounded by other forms of decreased sound tolerance such as hyperacusis or phonophobia. Given these results, we strongly recommend that all putative measures of misophonia or other sound tolerance disorder symptoms be demonstrated to correlate more strongly with other measures of the same symptom domain than with measures of phenomenologically different symptoms. Otherwise, research on misophonia risks conflating misophonia with more broadly defined decreased sound tolerance, potentially leading to incorrect conclusions about the most effective diagnostic/screening methods for misophonia or the overlap of misophonia with other sound tolerance disorders such as hyperacusis and phonophobia. Future research is, therefore, much needed to determine the most appropriate ways to psychometrically distinguish different forms of decreased sound tolerance from one another, particularly when using self-report questionnaires.

This investigation has a number of strengths, including a large and diverse sample of autistic and non-autistic adults; rigorous data-quality checks to ensure valid survey responses; clinical characterization that included additional measures of misophonia, hyperacusis, phonophobia, psychopathology, and somatic symptoms; confirmation of dimensionality with established and novel methods; sophisticated bifactor latent variable models with out-of-sample model fit assessment; and robust tests of differential item and test functioning across multiple subpopulations. However, it is not without limitations. Most notably, there was no interview-based “gold-standard” used to determine misophonia status, and we were therefore unable to report on the criterion-related validity or diagnostic efficiency (e.g., sensitivity, specificity, positive/negative predictive values) of the DVMSQ diagnostic algorithm in either sample. Future work is, thus, necessary to determine whether the DVMSQ algorithm is calibrated appropriately to screen for misophonia that rises to the level of clinical significance as judged by a trained clinician interviewer. In addition, the consensus definition-based diagnostic criteria used by our team were created after DVMSQ data were collected; consequently, the DVMSQ did not encompass all aspects of the condition mentioned in the consensus definition (e.g., “indirect” forms of avoidance such as altering others’ “triggering” behavior; Cowan et al., 2022). Future versions of the DVMSQ and other criterion-based misophonia screening tools should, therefore, be developed to fully capture the features of misophonia as reflected in the current diagnostic criteria or any more rigorously developed consensus criteria that are proposed in the literature. Another limitation of the current study was its cross-sectional design, as this precluded any analyses of the test-retest reliability of the DVMSQ total or subscale scores, the temporal stability of DVMSQ misophonia classification, or assessment of DIF across multiple administrations. As such, additional studies are needed to assess these properties of the measure, particularly if researchers are interested in using the DVMSQ to quantify change in misophonia symptoms due to treatments such as cognitive-behavioral therapy (Jager et al., 2021) or pharmacological interventions (Webb, 2022). Additional IRT-based psychometric analyses, such as determining the level of latent misophonia severity that can be measured precisely by the DVMSQ and validating scoring algorithms that differentially weight each item from the measure represent worthwhile future directions. Finally, despite promising data in the general population and independent autistic adults, the DVMSQ has not yet been validated for use in adolescents, autistic adults with intellectual disabilities, or other clinical populations of interest, and further research is warranted to determine whether this measure is appropriate to assess misophonia in these groups. In particular, given the frequent onset of misophonia symptoms in childhood or adolescence (Potgieter et al., 2019), there is a great need for screening tools in this age range, and we believe the readability of the DVMSQ makes it a strong candidate measure for potential further testing in younger age groups. Independent replication of the latent structure of the revised DVMSQ in general-population datasets would also be informative regarding the structure of the items in the context of the new screening item clarification text and the removal of two additional Likert items from the original scale.

## Conclusion

The DVMSQ is a novel self-report measure of misophonia symptoms and associated functional impairment designed to capture the core aspects of this disorder and to assign misophonia caseness according to a theory-based diagnostic algorithm. Based on initial psychometric testing of the DVMSQ in over 2,000 adult participants, the measure demonstrates a robust and replicable latent structure, adequate reliability and construct validity, and practically ignorable differential item and test functioning between autistic and non-autistic adults. The DVMSQ total score can be used as a global summary measure of misophonia symptoms and impairment, and separate symptom and impairment subscale scores are also available to investigate these two aspects of the misophonia construct. Despite encouraging preliminary psychometric data, further research is needed to independently validate these findings and extend them to other measurement properties (e.g., test-retest reliability and diagnostic efficiency) and respondent populations (e.g., adolescents, individuals with intellectual disability). However, in light of these initial data, we believe that the DVMSQ represents a promising measure of misophonia for use in research and clinical practice, particularly when assessing the features of misophonia seen in adults on the autism spectrum. As the field of misophonia research is rapidly growing and changing, additional revisions of this scale will undoubtedly be necessary as the very definition of misophonia is revised and updated to more accurately capture the lived experiences of individuals with this poorly understood disorder.

## Data availability statement

The names of the repository/repositories and accession number(s) can be found below: the general population (Prolific) dataset analyzed for this study can be downloaded from the Open Science Framework at https://osf.io/y6edb/. Approved researchers can obtain the SPARK (autistic adult) dataset described in this study by applying at https://base.sfari.org after a pre-specified embargo period.

## Ethics statement

The studies involving human participants were reviewed and approved by the Institutional Review Board at Vanderbilt University Medical Center. The patients/participants provided their written informed consent to participate in this study.

## Author contributions

ZW conceptualized and designed the study, wrote the initial draft of the DVMSQ, led data collection, analysis, and interpretation, and wrote the initial draft of the manuscript. TW and CC obtained funding for the project, assisted with study design and interpretation of findings, supervised all aspects of the project, and contributed to manuscript writing. All authors contributed to the article and approved the submitted version.

## Conflict of interest

ZW was received consulting fees from Roche and Autism Speaks. He also serves as a member of the autistic researcher review board of the Autism Intervention Research Network for Physical Health (AIR-P) and as a community partner for the Autism Care Network vanderbilt site. TW was the parent of an autistic adult. The remaining author declares that the research was conducted in the absence of any commercial or financial relationships that could be construed as a potential conflict of interest.

## Publisher’s note

All claims expressed in this article are solely those of the authors and do not necessarily represent those of their affiliated organizations, or those of the publisher, the editors and the reviewers. Any product that may be evaluated in this article, or claim that may be made by its manufacturer, is not guaranteed or endorsed by the publisher.
