# Supplementary material for: Psychometric validation of a brief self-report measure of misophonia symptoms and functional impairment: The duke-vanderbilt misophonia screening questionnaire
Source: Front Psychol. 2022 Jul 22;13:897901. doi: 10.3389/fpsyg.2022.897901 (PMC9355318; doi:10.3389/fpsyg.2022.897901)
Supplement: Supplementary file 1 [file Data_Sheet_1.docx]

Supplementary Material

**Supplementary Table 1.** Bifactor Graded Response Model Parameters for the DVMSQ Symptom Model in the Prolific Sample that Completed All DVMSQ Items (*n* = 833)

|  | ***a*_1_** | ***a*_2_** | ***a*_3_** | ***d*­_1_** | ***d*_2_** | ***d*_3_** | ***d*_4_** |
| --- | --- | --- | --- | --- | --- | --- | --- |
| **Item 1** | 2.13 | 0.99 | — | 5.78 | 3.52 | 0.49 | -2.44 |
| **Item 2** | 9.51 | 10.60 | — | 10.75 | 0.64 | -10.39 | -21.77 |
| **Item 4** | 0.87 | 0.44 | 0.50 | 1.42 | 0.54 | -0.96 | -2.61 |
| **Item 5** | 3.15 | — | 2.74 | 2.37 | 0.06 | -2.83 | -6.53 |
| **Item 6** | 2.01 | — | 0.95 | 2.75 | 1.13 | -0.92 | -2.75 |
| **Item 7** | 1.75 | 1.29 | — | -0.18 | -1.84 | -3.43 | -5.10 |
| **Item 8** | 2.25 | — | — | 0.64 | -0.68 | -2.46 | -4.65 |
| **Item 9** | 2.61 | — | — | 3.86 | 2.04 | -0.36 | -2.89 |
| **Item 10** | 1.26 | — | 0.35 | 0.17 | -0.47 | -1.59 | -2.89 |
| **Item 12** | 1.13 | — | 0.44 | 1.33 | -0.11 | -1.70 | -3.40 |

*Note.* The model assumes that all latent variables are orthogonal and have a standard normal distribution (i.e., *M* = 0, *SD* = 1) in the population. *a*_1_–*a*_3_ = slope parameters (higher values indicate stronger relationships with the latent variables [i.e., stronger factor loadings]); *d*_1_–*d*_4_ = intercept parameters (higher values indicate “less difficult” or more easily endorsed item categories).

**Supplementary Table 2.** Standardized Factor Loadings and Bifactor Statistical Indices for the DVMSQ Symptom Model in the Prolific Sample that Completed All DVMSQ Items (*n* = 833)

|  | **G** | **S_1_** | **S_2_** | ***h*^2^** | ***I-ECV*** |
| --- | --- | --- | --- | --- | --- |
| **Item 1** | 0.735 | 0.340 | — | 0.656 | 0.824 |
| **Item 2** | 0.663 | 0.739 | — | 0.986 | 0.446 |
| **Item 4** | 0.431 | 0.216 | 0.245 | 0.293 | 0.635 |
| **Item 5** | 0.699 | — | 0.607 | 0.858 | 0.570 |
| **Item 6** | 0.718 | — | 0.338 | 0.630 | 0.818 |
| **Item 7** | 0.634 | 0.467 | — | 0.620 | 0.648 |
| **Item 8** | 0.798 | — | — | 0.637 | 1.000 |
| **Item 9** | 0.838 | — | — | 0.702 | 1.000 |
| **Item 10** | 0.586 | — | 0.161 | 0.370 | 0.930 |
| **Item 12** | 0.539 | — | 0.210 | 0.335 | 0.869 |
|  |  |  |  |  |  |
| **Bifactor Coefficients:** | **G** | **S_1_** | **S_2_** |  |  |
| ω_T_/ω_S_ | 0.927 | 0.834 | 0.800 |  |  |
| ω_H_/ω_HS_ | 0.825 | 0.282 | 0.171 |  |  |
| *ECV* | 0.747 | 0.614 | 0.734 |  |  |

*Note.* Standardized factor loadings are based on a confirmatory full-information maximum likelihood factor model. G and S_1_/S_2_ refer to the general and specific factors, respectively. *h*^2^ = communality; ω_T_ = omega total (total sum score reliability); ω_S_ = omega subscale (subscale sum score reliability); ω_H_ = omega hierarchical (general factor saturation); ω_HS_ = omega total (subscale specific factor saturation); *ECV* = explained common variance for general factor (presented for each item, total score, and subscale).

**Supplementary Table 3.** Bifactor Graded Response Model Parameters for the DVMSQ Impairment Model in the Prolific Sample that Completed All DVMSQ Items (*n* = 833)

|  | ***a*_1_** | ***a*_2_** | ***d*_1_** | ***d*_2_** | ***d*_3_** | ***d*_4_** |
| --- | --- | --- | --- | --- | --- | --- |
| **Item 13** | 3.72 | — | -1.78 | -5.12 | -7.69 | -9.79 |
| **Item 14** | 2.18 | — | -0.82 | -2.82 | -4.63 | -6.53 |
| **Item 15** | 3.40 | — | -2.8 | -4.97 | -7.29 | -8.86 |
| **Item 16** | 3.06 | — | -3.22 | -5.02 | -6.62 | -8.47 |
| **Item 18** | 4.40 | 2.12 | -0.23 | -4.46 | -7.72 | -10.64 |
| **Item 19** | 6.88 | 3.91 | -0.35 | -7.35 | -11.78 | -16.31 |
| **Item 20** | 4.36 | 2.06 | -2.87 | -6.44 | -8.48 | -10.72 |

*Note.* The model assumes that all latent variables are orthogonal and have a standard normal distribution (i.e., *M* = 0, *SD* = 1) in the population. *a*_1_–*a*_2_ = slope parameters (higher values indicate stronger relationships with the latent variables [i.e., stronger factor loadings]); *d*_1_–*d*_4_ = intercept parameters (higher values indicate “less difficult” or more easily endorsed item categories).

**Supplementary Table 4.** Standardized Factor Loadings and Bifactor Statistical Indices for the DVMSQ Impairment Model in the Prolific Sample that Completed All DVMSQ Items (*n* = 833)

|  | **G** | **S_1_** | ***h^2^*** | ***I-ECV*** |
| --- | --- | --- | --- | --- |
| **Item 13** | 0.909 | — | 0.827 | 1.000 |
| **Item 14** | 0.788 | — | 0.621 | 1.000 |
| **Item 15** | 0.894 | — | 0.800 | 1.000 |
| **Item 16** | 0.874 | — | 0.764 | 1.000 |
| **Item 18** | 0.851 | 0.409 | 0.892 | 0.812 |
| **Item 19** | 0.850 | 0.483 | 0.956 | 0.756 |
| **Item 20** | 0.853 | 0.403 | 0.889 | 0.818 |
|  |  |  |  |  |
| **Bifactor Coefficients:** | **G** | **S_1_** |  |  |
| ω_T_/ω_S_ | 0.958 | 0.944 |  |  |
| ω_H_/ω_HS_ | 0.905 | 0.146 |  |  |
| *ECV* | 0.902 | 0.794 |  |  |

*Note.* Standardized factor loadings are based on a confirmatory full-information maximum likelihood factor model. G and S_1_ refer to the general and specific factors, respectively. *h*^2^ = communality; ω_T_ = omega total (total sum score reliability); ω_S_ = omega subscale (subscale sum score reliability); ω_H_ = omega hierarchical (general factor saturation); ω_HS_ = omega total (subscale specific factor saturation); *ECV* = explained common variance for general factor (presented for each item, total score, and subscale).

**Supplementary Table 5.** Standardized Factor Loadings and Bifactor Statistical Indices for the DVMSQ Full Model in the Prolific Sample that Completed All DVMSQ Items (*n* = 833)

|  | **G** | **S_1_** | **S_2_** | **S_3_** | **S_4_** | ***h^2^*** | ***I-ECV*** |
| --- | --- | --- | --- | --- | --- | --- | --- |
| **Item 1** | 0.704 | 0.379 | — | — | — | 0.639 | 0.776 |
| **Item 2** | 0.653 | 0.727 | — | — | — | 0.955 | 0.446 |
| **Item 4** | 0.406 | 0.256 | 0.300 | — | — | 0.320 | 0.514 |
| **Item 5** | 0.720 | — | 0.537 | — | — | 0.807 | 0.643 |
| **Item 6** | 0.726 | — | 0.352 | — | — | 0.651 | 0.809 |
| **Item 7** | 0.635 | 0.481 | — | — | — | 0.635 | 0.635 |
| **Item 8** | 0.804 | — | — | — | — | 0.647 | 1.000 |
| **Item 9** | 0.833 | — | — | — | — | 0.694 | 1.000 |
| **Item 10** | 0.603 | — | 0.147 | — | — | 0.385 | 0.944 |
| **Item 12** | 0.557 | — | 0.199 | 0.305 | — | 0.443 | 0.701 |
| **Item 13** | 0.615 | — | — | 0.673 | — | 0.831 | 0.455 |
| **Item 14** | 0.493 | — | — | 0.598 | — | 0.601 | 0.405 |
| **Item 15** | 0.547 | — | — | 0.701 | — | 0.791 | 0.379 |
| **Item 16** | 0.471 | — | — | 0.753 | — | 0.789 | 0.281 |
| **Item 18** | 0.656 | — | — | 0.576 | 0.367 | 0.896 | 0.480 |
| **Item 19** | 0.662 | — | — | 0.567 | 0.441 | 0.954 | 0.459 |
| **Item 20** | 0.649 | — | — | 0.581 | 0.356 | 0.886 | 0.476 |
|  |  |  |  |  |  |  |  |
| **Bifactor Coefficients:** | **G** | **S_1_** | **S_2_** | **S_3_** | **S_4_** |  |  |
| ω_T_/ω_S_ | 0.977 | 0.812 | 0.787 | 0.914 | 0.587 |  |  |
| ω_H_/ω_HS_ | 0.756 | 0.295 | 0.187 | 0.457 | 0.149 |  |  |
| *ECV* | 0.586 | 0.584 | 0.723 | 0.443 | 0.471 |  |  |

*Note.* Standardized factor loadings are based on a confirmatory full-information maximum likelihood factor model. G and S_1–4_ refer to the general and specific factors, respectively. *h*^2^ = communality; ω_T_ = omega total (total sum score reliability); ω_S_ = omega subscale (subscale sum score reliability); ω_H_ = omega hierarchical (general factor saturation); ω_HS_ = omega total (subscale specific factor saturation); *ECV* = explained common variance for general factor (presented for each item, total score, and subscale).

**Supplementary Table 6.** Standardized Factor Loadings and Bifactor Statistical Indices for the DVMSQ Full Model in the SPARK Sample that Completed All DVMSQ Items (*n* = 647)

|  | **G** | **S_1_** | **S_2_** | **S_3_** | **S_4_** | ***h^2^*** | ***I-ECV*** |
| --- | --- | --- | --- | --- | --- | --- | --- |
| **Item 1** | 0.651 | 0.314 | — | — | — | 0.522 | 0.812 |
| **Item 2** | 0.609 | 0.756 | — | — | — | 0.942 | 0.394 |
| **Item 4** | 0.481 | 0.309 | -0.033 | — | — | 0.328 | 0.706 |
| **Item 5** | 0.763 | — | 0.328 | — | — | 0.690 | 0.844 |
| **Item 6** | 0.693 | — | 0.529 | — | — | 0.760 | 0.632 |
| **Item 7** | 0.575 | 0.519 | — | — | — | 0.600 | 0.551 |
| **Item 8** | 0.708 | — | — | — | — | 0.501 | 1.000 |
| **Item 9** | 0.800 | — | — | — | — | 0.640 | 1.000 |
| **Item 10** | 0.610 | — | 0.051 | — | — | 0.374 | 0.993 |
| **Item 12** | 0.574 | — | 0.113 | 0.226 | — | 0.393 | 0.838 |
| **Item 13** | 0.553 | — | — | 0.738 | — | 0.851 | 0.359 |
| **Item 14** | 0.436 | — | — | 0.735 | — | 0.731 | 0.260 |
| **Item 15** | 0.513 | — | — | 0.684 | — | 0.731 | 0.360 |
| **Item 16** | 0.431 | — | — | 0.684 | — | 0.654 | 0.284 |
| **Item 18** | 0.634 | — | — | 0.540 | 0.421 | 0.870 | 0.462 |
| **Item 19** | 0.640 | — | — | 0.506 | 0.492 | 0.907 | 0.451 |
| **Item 20** | 0.632 | — | — | 0.470 | 0.451 | 0.823 | 0.485 |
|  |  |  |  |  |  |  |  |
| **Bifactor Coefficients:** | **G** | **S_1_** | **S_2_** | **S_3_** | **S_4_** |  |  |
| ω_T_/ω_S_ | 0.957 | 0.838 | 0.767 | 0.913 | 0.648 |  |  |
| ω_H_/ω_HS_ | 0.740 | 0.353 | 0.077 | 0.474 | 0.218 |  |  |
| *ECV* | 0.567 | 0.567 | 0.784 | 0.417 | 0.465 |  |  |

*Note.* Standardized factor loadings are based on a confirmatory full-information maximum likelihood factor model. G and S_1–4_ refer to the general and specific factors, respectively. *h*^2^ = communality; ω_T_ = omega total (total sum score reliability); ω_S_ = omega subscale (subscale sum score reliability); ω_H_ = omega hierarchical (general factor saturation); ω_HS_ = omega total (subscale specific factor saturation); *ECV* = explained common variance for general factor (presented for each item, total score, and subscale).
