# Supplementary material for: Psychometric validation of a brief self-report measure of misophonia symptoms and functional impairment: The duke-vanderbilt misophonia screening questionnaire
Source: Front Psychol. 2022 Jul 22;13:897901. doi: 10.3389/fpsyg.2022.897901 (PMC9355318; doi:10.3389/fpsyg.2022.897901)
Supplement: Supplementary file 2 [file Data_Sheet_2.pdf]

Duke-Vanderbilt Misophonia Screening Questionnaire

S1. Are there specific sounds that you are <u>extremely</u> bothered by, <u>even if they are not loud</u>? ☐ **Yes** ☐ **No**

Examples include: chewing, slurping, crunching, throat clearing, finger tapping, foot shuffling, keyboard tapping, rustling, nasal sounds, pen clicking, appliance humming, clock ticking, and animal sounds.

These sounds should cause <u>significant emotional distress</u> (e.g., extreme irritation, anger, disgust, rage, anxiety, or panic). Do NOT count sounds that bother you <u>only</u> because you find them too loud or physically painful.

<u>If you respond "No" to this question, you have finished the questionnaire. Do not answer the other questions.</u>

S2. Please list the sounds that you are extremely bothered by, even when they are soft.

When you are exposed to the bothersome sounds listed above, how often do you experience...	Never	Rarely	Some-times	Often	Very often
**1. Intense feelings of irritation or annoyance?**	☐	☐	☐	☐	☐
**2. Feelings of anger or rage?**	☐	☐	☐	☐	☐
**3. Feelings of fear or panic?**	☐	☐	☐	☐	☐
**4. Feelings of disgust?**	☐	☐	☐	☐	☐
**5. Urges to run away from the sound?**	☐	☐	☐	☐	☐
**6. Urges to cover your ears or block out the sound in some other way?**	☐	☐	☐	☐	☐
**7. Urges to lash out violently at the person or object making the sound?**	☐	☐	☐	☐	☐
**8. Feeling like you cannot control your response to the sound?**	☐	☐	☐	☐	☐
**9. Difficulty focusing on anything except the sound?**	☐	☐	☐	☐	☐
**10. Some sort of immediate physical response?** *(e.g., tensing of muscles, heart racing, warmth, tingling, pain, or tightening of stomach)* **Please describe:** ______	☐	☐	☐	☐	☐

	Never	Rarely	Some-times	Often	Very often
**11. How often do you avoid situations where you may potentially hear these bothersome sounds?**	☐	☐	☐	☐	☐

<u>Please see reverse side for additional questions</u>

DVMSQ v1.5: Copyright © 2022 Zachary J. Williams. No permission required to reproduce, adapt, modify, translate, display, or distribute.

In the past 7 days, how much did your sound sensitivities interfere with...	Not at all	A little bit	A moderate amount	Very much	An extreme amount
12. Your ability to interact with other people?	☐	☐	☐	☐	☐
13. Your ability to be productive at work or school?	☐	☐	☐	☐	☐
14. Your ability to take care of your household responsibilities?	☐	☐	☐	☐	☐
15. Your ability to participate in community activities (for example, festivities, religious, or other activities)?	☐	☐	☐	☐	☐

	Not at all	A little bit	A moderate amount	Very much	An extreme amount
16. To what degree have your sound sensitivities negatively impacted your mental or emotional health?	☐	☐	☐	☐	☐
17. To what degree do you believe that your sound sensitivities have created problems for you?	☐	☐	☐	☐	☐
18. To what degree do you believe that your sound sensitivities have made your entire life worse?	☐	☐	☐	☐	☐

Trigger List (Optional)

Which of the following <u>bother you much more intensely</u> than they do most other people?

People making mouth sounds while eating or drinking (e.g., chewing, crunching, slurping).	☐
People making nasal/throat sounds (e.g., sniffing, sneezing, nose-whistling, coughing, throat-clearing).	☐
People making mouth sounds when not eating (e.g., making the "tsk" sound, heavy breathing, snoring, whistling).	☐
People making repetitive sounds (e.g., typing, tapping nails on table, pen clicking, writing, construction work, using machinery).	☐
Rustling or tearing objects (e.g., paper, plastic).	☐
Speech sounds (e.g., "p" sounds, hissing "s" sounds, someone speaking with a lisp, high-pitched voices).	☐
Body or joint sounds (e.g., snapping fingers, cracking joints, jaw clicking).	☐
Rubbing sounds (e.g., hands on pants, hands against one another, styrofoam rubbing together).	☐
Stomping or loud walking (e.g., heels clicking, flip flops, etc.).	☐
Muffled sounds (e.g., voices separated by a wall, TV/music in another room).	☐
People talking in the background (e.g., phone calls in public, many people talking at once).	☐
Repetitive or continuous sounds not made by a person (e.g., clock ticking, air conditioner humming, water running).	☐
Animals making repetitive sounds (e.g., licking, chirping, barking, eating, drinking).	☐
Seeing someone making or about to make a sound that bothers you, even if you can't hear it (e.g., seeing someone reach into a bag of chips, seeing someone eating on TV with the volume off).	☐

<u>On average</u> across <u>ALL bothersome sounds</u>, rate how often you are bothered by these sound/sounds.

Once per month or less ☐	2-3 times per month ☐	1-3 times per week ☐	4-7 times per week ☐	2-5 times per day ☐	6 or more times per day ☐

DVMSQ v1.5: Copyright © 2022 Zachary J. Williams. No permission required to reproduce, adapt, modify, translate, display, or distribute.

DVMSQ Scoring Instructions

The **Duke-Vanderbilt Misophonia Screening Questionnaire (DVMSQ)** is an 18-item self-report measure of symptoms associated with the newly described condition of misophonia.

Scoring rules for the DVMSQ items are detailed below. Respondents who endorse respond "Yes" to an initial screening question are asked to complete 18 additional Likert items, which are each measured on 5-point Likert scales from 0–4. **Total scores range from 0–68, with higher scores indicating a higher degree of misophonia severity.** Specific DVMSQ cutoff scores have not yet been validated against a clinical diagnosis of misophonia, although a theory-based diagnostic algorithm is available for use. Additionally, the total score on the DVMSQ Likert items can be used as a dimensional misophonia severity scale in research and clinical practice.

Screening item (S1): (*"Are there specific sounds that you are extremely bothered by, even if they are not loud?"*)

- **Yes** = 1 (continue on to remainder of questionnaire)
- **No** = 0 (questionnaire is over)

If score of 0 on screening item (S1), questionnaire is finished. Total score is 0.

DVMSQ Likert Item Scoring

Symptom Frequency Items (Items 1–11; Responses: *"Never"* to *"Very often"*)

- **Never** = 0
- **Rarely** = 1
- **Sometimes** = 2
- **Often** = 3
- **Very often** = 4

Interference Items (Items 12–18; Responses: *Not at all* to *"An extreme amount"*)

- **Not at all** = 0
- **A little bit** = 1
- **A moderate amount** = 2
- **Very much** = 3
- **An extreme amount** = 4

DVMSQ Total Score: Sum of all 0–4 item scores for **items 1, 2, and 4–18 (range: 0–68). [item 3 does not contribute to this score]**
DVMSQ Symptom Score: Sum of all 0–4 item scores for **items 1, 2, and 4–11 (range: 0–40).**
DVMSQ Impairment Score: Sum of all 0–4 item scores for **items 12–18 (range: 0–28).**

DVMSQ Theory-based Diagnostic Algorithm (Williams et al., 2022)

(*based on combination of Revised Amsterdam Criteria and Misophonia Consensus Definition*)

Misophonia Criterion	Operationalization
A. Presence of one or more commonplace "trigger" sounds[a] that reliably elicit intense and inappropriate emotional responses, irrespective of sound intensity or perceived loudness.	**Item S1 [Screening] = Yes**
B. Trigger sounds reliably[b] evoke feelings of extreme irritation, anger, rage and/or disgust[c] that are clearly excessive, unreasonable, or out of proportion to the circumstances (whether or not the individual recognizes them as such).	One or more of the following<br>- **Item 1 [Irritation] ≥ Often**<br>- **Item 2 [Anger/Rage] ≥ Often**<br>- **Item 4 [Disgust] ≥ Often**
C. The individual actively avoids[d] situations or activities that include trigger sounds, endures these situations with intense discomfort, or needs to block out potential trigger sounds (e.g., using earplugs, music, or white noise) to cope with these situations.	One or more of the following<br>- **Item 11 [Avoidance] ≥ Sometimes**<br>- **Item 5 [Urge to run away] ≥ Often**<br>- **Item 6 [Urge to cover ears] ≥ Often**
D. If unable to avoid trigger sounds or stop them from occurring, the individual experiences a significant loss of self-control, potentially resulting in emotional outbursts or other extreme reactions (e.g., yelling/screaming, running out of the room, panic attacks, rarely physical aggression)	One or more of the following<br>- **Item 8 [Lack of Control] ≥ Sometimes**<br>- **Item 7 [Urge to be violent] ≥ Sometimes**
E. The emotional reactions to trigger sounds are persistent, typically lasting for 6 months or more. Specific triggers do not need to remain constant over this period, but at least one trigger sound must meet both criteria A and B at all times over the preceding 6-month period.	Not assessed by DVMSQ<br>- Assumed to be true if all other criteria are satisfied.
F. Emotional reactions to trigger sounds and/or avoidance of these sounds cause clinically significant distress or impairment in social, occupational, or other important areas of functioning.	Two or more of the following<br>- **Item 12 [Social] ≥ Moderate**<br>- **Item 13 [Occupational] ≥ Moderate**<br>- **Item 14 [Household] ≥ Moderate**<br>- **Item 15 [Community] ≥ Modrate**<br>- **Item 16 [Mental Health] ≥ Moderate**<br>- **Item 17 [Global Problems] ≥ Moderate**<br>- **Item 18 [Life Affected] ≥ Moderate**

Clinically significant misophonia is defined as meeting all **criteria A–F** (i.e., supra-threshold symptoms AND impairment)

Sub-clinical misophonia is defined as meeting **criteria A–D but not criterion F** (i.e., supra-threshold symptoms but NO impairment)

DVMSQ v1.5: Copyright © 2022 Zachary J. Williams. No permission required to reproduce, adapt, modify, translate, display, or distribute.
